# Stochastic phenotypic switching arises in response to directional selection in experimentally evolved multicellular yeast

**DOI:** 10.1038/s42003-025-09414-9

**Published:** 2025-12-27

**Authors:** Beatriz Baselga-Cervera, Nahui Olin Medina-Chávez, Noah Gettle, Michael Travisano

**Affiliations:** 1 Department of Ecology, Evolution and Behavior, University of Minnesota, St Paul, MN USA; 2 Minnesota Center for Philosophy of Science, University of Minnesota, Minneapolis, MN USA; 3 The Biotechnology Institute, University of Minnesota, St Paul, MN USA; 4 Department of Biological Sciences, University of Denver, Denver, CO USA

**Keywords:** Experimental evolution, Microbial genetics

## Abstract

Phenotypic diversity in genetically homogenous populations has increasingly been recognized as potentially adaptive even under constant conditions. The origins of adaptive differentiation during major evolutionary transitions, such as the evolution of multicellularity and eusociality, are generally thought to arise from pre-existing stochastic and plastic phenotypic heterogeneity. Here, we characterize phenotypic diversity in isogenic populations of experimentally-evolved multicellular yeast, *Saccharomyces cerevisiae*. Our results show support for a bistable system maintained across different growth conditions, consisting of two distinct morphotypes: large multicellular clusters and smaller ancestral-like clusters consisting of one to a few cells. This bistable system arises as a pleiotropic consequence of a loss-of-function mutation in ACE2 that generates the selected large multicellular clusters concomitantly with the metabolically distinct alternative small clusters phenotype. Time-course assays and mathematical modeling indicate that small clusters phenotype arise directly from the growth of derived multicellular phenotypic individuals, consistent with stochastic phenotypic switching. Furthermore, we found significant differences in gene expression between the two different morphotypes, which cannot be readily explained by microenvironmental variation and instead suggest that the morphotypes occupy distinct growth states. Our study offers insights into how stochastic phenotypic switching can influence evolution by maintaining biological diversity in nascent multicellularity.

## Abstract (web summary)

A study of experimentally evolved multicellular yeast reveals distinct phenotypic subpopulations driven by a mutation in ACE2, which maintain phenotypic diversity and suggest that stochastic switching influences early multicellularity evolution.

## Introduction

Evolutionary responses to directional selection are foundational examples of evolution. Well-known examples include coloration of the peppered moth^1^, beak sizes in Darwin’s finches^2^, and antibiotic resistance in bacteria^3^. These and many other examples have led to a well-developed theoretical framework for predicting adaptive evolution (e.g., refs. ^4,5^). This theory has utility across many fields, including vaccine development^6^, agriculture^7^, and conservation biology^8^. Quantitative evolutionary predictions, however, remain challenging^9^ despite the many examples of directional selection. This is because evolutionary responses involve a mix of stochastic genetic events, such as mutation, recombination, and drift, on top of complex genetic landscapes of linkage, epistasis, and pleiotropy^10^.

The repeated evolutionary origins of multicellularity are examples of major evolutionary transitions driven by directional selection^11^. Much of the transformative consequences of multicellularity build upon cellular differentiation, which opened new avenues for adaptation and is most clearly exemplified in plants, animals, and fungi. Theoretical models typically posit that differentiation evolves from pre-existing environmentally induced (phenotypic plasticity) or stochastically generated phenotypic heterogeneity in unicellular precursors; however, the selective benefits of such epigenetic-based variation are uncertain. In recent years, there has been increasing emphasis on the role of plasticity in the evolution of differentiation and development systems^12^, particularly in unicellular ancestors^13,14^. But the implications of phenotypic heterogeneity in maintaining phenotypic diversity and the evolvability of the alternative phenotypes have been overlooked. Unicellular plasticity is often in response to resource availability or can evolve as a bet-hedging strategy in unpredictable environments, altering growth-associated gene expression, and would not necessarily provide a fitness benefit in a multicellular context. In this study, we investigate the phenotypic consequences following directional selection for increased size. Concretely, how phenotypic heterogeneity can arise in isogenic populations of experimentally evolved multicellular yeast, *Saccharomyces cerevisiae*.

In previous studies, multicellular *S. cerevisiae* phenotypes rapidly evolved during experimental evolution for settling^15–18^. Multicellular individuals are 20 – 100 times the size of their unicellular ancestors, and subsequent selection yields even greater size increases^15–18^. Simultaneously, population size distributions of experimentally evolved multicellular clonal genotypes display bimodal size distributions (see refs. ^19–21^) that include individuals in the same size range as the unicellular ancestors. While the total biomass in a population of the small size class is modest (<2%), approximately half of the population consists of small individuals. These small-sized individuals, however, were not observed during short-term microscopic analyses of phenotype^15,22^, suggesting variability in the appearance of the small size class.

There is evidence that bimodality or multimodality is a common pattern in populations of microorganisms^23,24^. It is frequently linked to a spreading-the-risk strategy that confers ecological advantages^11–14^ and is an evolvable trait that can potentiate adaptation in new environments^25,26^. Certain genetic regulatory circuits and stochastic gene expression^23,27,28^ can generate the bifurcation of microbial isogenic populations into morphotypes, resulting in the bistable expression of two distinct stable coexisting phenotypic subpopulations. In settling-selected *S. cerevisiae*, multicellularity evolves due to loss-of-function (LOF) mutations that impact late-stage cell division. Multiple studies have recently observed that LOF mutations facilitate adaptive evolution^29,30^. In part, this may be because they can occur through multiple mutational events, and therefore, occur at a high frequency^31^, reducing stochasticity and waiting times for mutations. LOF mutations potentially serve as “major” minor mutations^32^ from which significant phenotypic or fitness changes can arise. Just as mutations in developmental pathways can have profound phenotypic consequences in multicellular organisms^33^, LOF mutations at regulatory loci can have diverse pleiotropic consequences and thereby generate phenotypic variation^34^, including effects on cell division. While several studies have addressed the evolutionary role of cells switching between phenotypic states^35,36^, a key question remains whether stochastic variation or/and phenotypic plasticity drives heterogeneity in ways that shape biological diversity across a profound innovation such as the evolutionary transition from single cells to multicellular forms.

Here, we show the emergence of multiple coexisting phenotypes within isogenic multicellular yeast genotypes. Evolutionary-derived *S. cerevisiae* populations consisted of two distinct numerically equivalent phenotypic size classes: large multicellular clusters and smaller ancestral-like clusters consisting of one to a few cells. Follow-up experiments validate that single genotypes generate the two distinct phenotype size classes across different environments, both under shaking and non-shaking conditions, and that the phenotypic heterogeneity is maintained over multiple cycles of growth. This pattern of a bimodal size distribution consisting of two distinct morphotypes is a consequence of LOF at the ACE2 locus, a gene that regulates the separation of mother-daughter cells after budding. We next formulated a mathematical model for the phenotypic distribution resulting from ACE2 LOF. Time-course assays and mathematical modeling imply that the transition between stages follows a phenotypic switching pattern. Using genome-wide transcriptional analysis in constructed strains, we identify the expression changes that distinguish the two coexisting phenotypic classes within isogenic multicellular yeast genotypes.

## Results

### Multicellular, large and small ancestral-like clusters are present in isogenic-derived genotypes

Our observations under the microscope of small clusters suggest that the evolved settling-selected populations comprise two subpopulations. To determine whether the evolved yeast growth involves two underlying groups, we conducted a series of phenotypic assays with different evolved isogenic strains. We previously measured the phenotypic size distributions of the unicellular WT (Y55 strain) and two derived multicellular strains (C1W8.1 and C1W8.2 strains) originally from the same population selected for settling ability^15^.

**Fig. 1:**
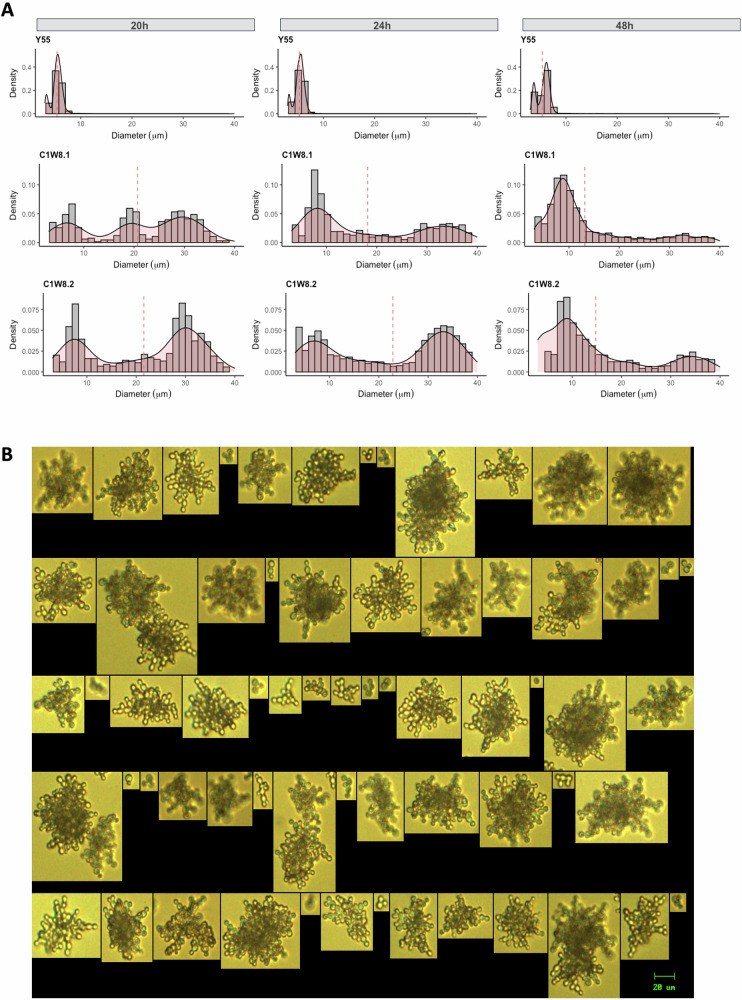
Bimodal size distributions in evolved isogenic multicellular strains. Multicellular-derived populations can generate two distinct phenotypes: smaller ancestral-like and larger multicellular clusters. Population distributions show support for a bistable system. **A** Each diameter distribution (µm) depicts the population density of whole populations after vortexing—note different *y*-axis limits—after 20, 24, and 48-h growth in YPD media under shaken conditions of one out of five independent isolates per ancestral (Y55) and multicellular-derived (C1W8.1 and C1W8.2) strains. Vertical lines represent the population’s mean values (see Fig. S2 for particle counter distributions of all the isolates). **B** Particles imaged obtained with FlowCam imaging microscopy from one isolate of the C1W8.2 evolved strain at 24-h growth. Images are representative of the whole well-mixed population.

Population size distributions of the derived genotypes at 24-h growth display bimodal size distributions under the experimental conditions they evolved (Fig. 1A, particle counter population’s diameter (µm) descriptive statistics and multimodality test of five independent isolates per strain at 24-h growth in Table S1). One of the subpopulations was composed of small-sized propagules (from 3 to 13 μm), the small clusters subgroup, and the other subpopulations comprised large individuals (>13 μm), the large clusters subgroup. We quantified both subpopulations by fitting a two-Gaussian distribution model (see a two-component normal mixture model analysis example in Fig. S1). For all multicellular strains at 24-h growth, approximately half of the counted particles were small clusters (Table S2).

Additionally, we assessed the population’s distributions after log phase at 12-h growth, 4 h before the normal transfer regime during early post-diauxic phase (20-h growth), and at the stationary phase (48-h growth). In the WT, we observe similar size distributions at 20-h and 24-h growth, and an increase in size at 48-h (Figs. 1A and S2). In the multicellular-derived genotypes, at 12-h growth, populations presented weak bimodal distributions, suggesting growth in terms of cell division (Fig. S3). At 20-h growth, populations presented multimodal distributions with two peaks of multicellular clusters that are at a lower end of the size range compared to the 24-h distributions. This indicates division at the large clusters level (Fig. 1A). At 48-h growth, most of the population is represented by small clusters (Fig. 1A). Increases in propagules of the alternative small phenotype suggest that they are produced by either phenotype between 24 and 48 h.

By FlowCam Imaging analysis, we visually confirmed the presence of phenotypes consisting of one to a few cells, the small clusters subgroup (Figs. 1B and S4). Again, we identified a small cluster subgroup composed of one to a few cells that represents 15–25% of the population (Figs. 1B and S4). Note that for this imaging analysis, the lower threshold for multicellular populations is 5 μm. Therefore, a fraction of the population, mainly composed of cells between 3 and 5 μm, was not measured.

The observed distributions support a bistable system. Across multicellular-derived strains at different growth times, we observed two distinct phenotypic subpopulations: the small size subgroup composed of one to a few cells, hereon referred to as small clusters, and the large subgroup comprising large clusters.

### Phenotypic heterogeneity is maintained across experimental conditions

**Fig. 2:**
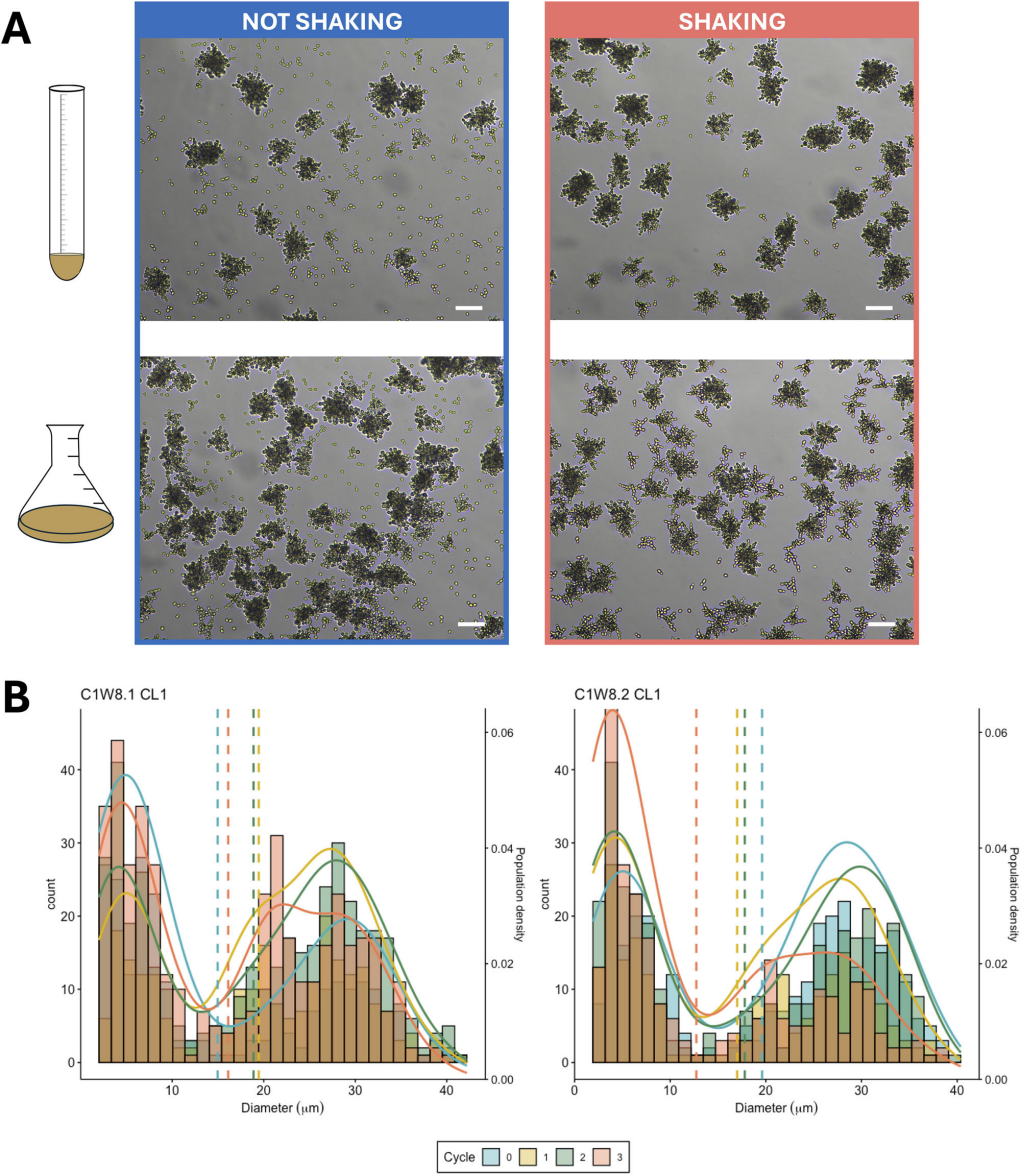
Phenotypic heterogeneity is maintained regardless of environmental perturbations or genotype. A bistable system, composed of smaller ancestral-like and large multicellular cluster phenotypes, is maintained across all the different environmental conditions tested. **A** Microphotographs of C1W8.1 evolved strain at 24-h growth in the original culture conditions—culture tubes with 10 ml of media—and one of the other tested culturing conditions—50 mL Erlenmeyer flasks with 30 mL of YPD media—under no shaking and shaking at 250 rpm (see Fig. S5 for values of all the strains by experimental condition). Small clusters were present in all conditions and increased in static conditions. **B** Cultures initiated with small clusters present bimodal distributions, as were the cultures from which the small clusters were obtained. Particle size distributions of an isolate per evolved strain (all colony lineages (CL) in Fig. S6) over a series of plating from the top after gravitational selection (selection cycles). Vertical lines indicate the particle’s mean diameter (μm), and colors denote the number of selection cycles.

We sought to understand if the physical environment is driving the observed phenotypic heterogeneity. To test the effect of physical forces—shaking—and culturing conditions, we compared isogenic isolates in different experimental conditions after 24 h growth (Fig. 2A). We found significant differences in the ratio of smaller ancestral-like and large multicellular clusters when comparing culturing conditions (two-way ANOVA *F*(2) = 14.57, *p* < 0.001, Fig. 2B, Table S3). Small clusters significantly increased in frequency when isolates were grown without shaking across the different environmental conditions and strains (two-way ANOVA *F*(1) = 374.68, *p* < 0.001, Fig. 2A, Table S3, Fig. S5). This observation can be explained by two effects: first, shaking conditions increase mixing of dissolved oxygen and nutrients influence cell growth and potentially favor large clusters growth, and second, the production of propagules of the smaller alternative phenotype is favored in static environments.

To evaluate the persistence of the observed phenotypic heterogeneity in populations derived from small clusters, we plated from the top fraction of the population after gravitational selection and picked random colonies. We hypothesize that small clusters generate bimodal population distributions similar to those from the populations from which they are derived. As expected, all selected colonies of the evolved C1W8.1 and C1W8.2 strains generate bimodal distributions. The bimodal distributions are maintained over three cycles of selecting colonies from the top (Fig. 2B). We did, however, observe significant differences in mean diameter (μm) over selection cycles (Kruskal–Wallis test, *p* < 0.01, Fig. 2B, statistical analyses in Table S4), indicating a decrease in size of the multicellular clusters subgroup (see the two-component normal mixture model analyses Table S5 and Fig. S6). However, density distributions are less than 25% dissimilar, maintaining the bimodal distribution (*η* similarity ranging from 0.74–0.76 to 0.97 in C1W8.1 and C1W8.2, Fig. S7).

The bistable system is maintained across all environmental conditions tested. Notably, static culture conditions promote a shift toward small ancestral-like clusters rather than inhibit it. This is in opposition to the expectation that the smaller clusters are more commonly generated due to increased shear forces associated with shaking.

### A recessive LOF mutation at the ACE2 locus generates observed phenotypic heterogeneity

We investigated the role of the recessive LOF mutation at the ACE2 locus in the observed phenotypic heterogeneity. The two genetically constructed multicellular strains (*ace2*Δ *missense* and ace*2*Δ *knockout* strains) resulted from transforming the *ace2Δ missense* causal allele, identified in both derived strains, and from knocking out the ACE2 gene into the WT Y55 genetic background.

**Fig. 3:**
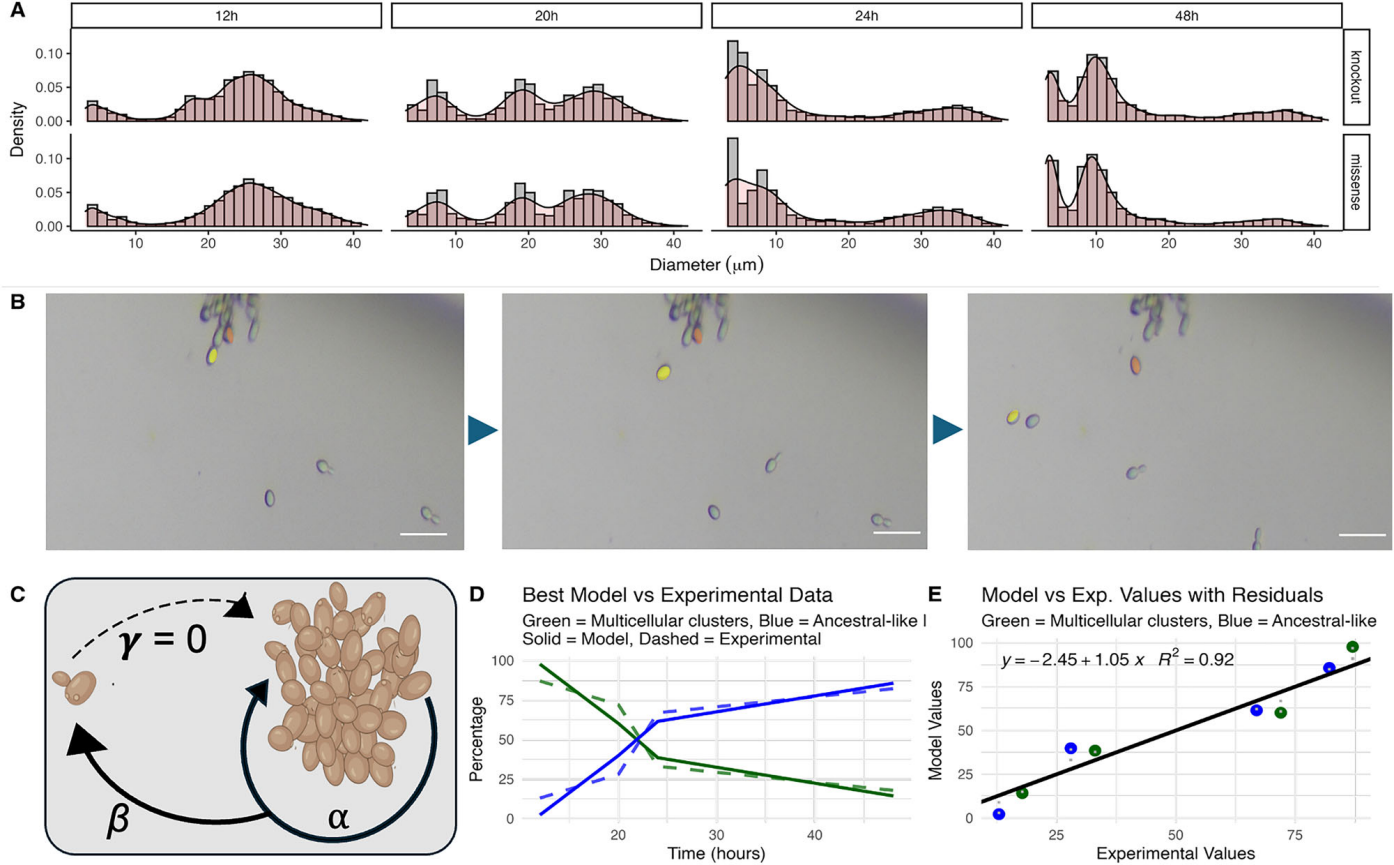
LOF mutation at the ACE2 locus enables the expression of two distinct phenotypic subpopulations. Time-course assays and mathematical modeling predictions conform to a model in which the small ancestral-like clusters are produced by the large multicellular clusters and are the result of a transition between stages after the large clusters reach a threshold. **A** We observed phenotypic heterogeneity in particle size distribution in the two constructed multicellular strains (*ace2Δ knockout* and *ace2Δ missense*) over a 48-h growth cycle. **B** A time-lapse sequence captures two instances of single-cell propagules (ancestral-like), highlighted in yellow and orange, respectively, separating from a large multicellular cluster of the C1W8.1-derived strain (see Movie S1). Scale bars: 10 μm. **C** Phenotypic dynamics of the constructed populations where smaller clusters only derive at a rate (*β*) from large multicellular clusters. **D** Phenotypic percentages over time and **E** residuals standard deviation plots represent the best-fit model (inset: *α* = 1, *β* = 0.005, *γ* = 0, and steepness (*k*) = 0.001) plotted with the experimental data. Our differential equations mathematical model accurately predicts the phenotypic dynamics.

Phenotypic assays of constructed strains recapitulate the bimodal size distribution observed in evolved multicellular lines. Both constructed strains show the same phenotypic expression as derived strains: size distributions composed of the small ancestral-like and large multicellular clusters (Fig. 3A). At 12 h growth, populations were bimodal, and large clusters were present but smaller in size, consistent with ongoing growth and division within multicellular clusters. At 20-h growth, population distributions are tri-modal and mainly composed of large clusters, indicating division at the large cluster level. Small clusters increase in frequency by 24-h growth, representing most of the bimodal populations at 48-h growth (Fig. 3A). Moreover, individual colonies randomly picked after plating the top fraction of *ace2Δ knockout* and *ace2Δ missense* populations after gravitational selection produce bimodal distributions. These bimodal distributions are like those of the populations from which they are derived and exhibit high pairwise overlap ranging from 0.54 to 0.97 (see size particle distributions and η for all pairwise comparisons, Fig. S8). The observed increase in the small clusters over time suggests that they are produced by large clusters (Fig. 3A).

Phenotypic switching events dynamics were captured in both derived and constructed via time-lapse microscopy. Large multicellular clusters undergo fragmentation into propagules and the generation of small ancestral-like propagules after ~17 h growth (Fig. 3B, Movie S1: C1W8.1-derived strain, and Movie S2: *ace2Δ knockout* constructed strain). Upon transfer to fresh media, small propagules undergo division for 2–3 h, after which cellular division halts for the rest of the observation period (see Movie S3). These observations support our model that small clusters are derived from large clusters once a switch threshold induces the transition between stages.

This result is mathematically tractable by modeling the phenotypic dynamics over time (*t*) of the whole populations, where small clusters (*U*) are produced at a rate ($\beta$) from large clusters (*M*), and accumulate over time. Large cluster (*M*) population growth, defined as cluster fragmentation into two clusters, can be calculated as *M* growth rate coefficient (*α*) by its doubling time as log_2_ (*M*), where *η*(*t*) represents random fluctuations in *M* growth. In practice, *U* is produced only in small amounts until the early post-diauxic growth phase (~20-h growth, see Fig. 3A, B), so we introduce a transition between stages: the probability that a stochastic switch occurs at *t* is given by a sigmoid function (*S* (*t*)). The probability that *U* transitions into *M* over *t* is represented by *γ*. We therefore can describe the phenotypic dynamics over time by means of the following differential equations (see supplemental materials for a detailed description of the model):

1 $$\frac{{dM}}{{dt}}=\alpha {log }_{2}\left(M\right)+\eta (t)+\gamma U$$

2 $$\frac{{dU}}{{dt}}=S(t)\beta M-\gamma U$$

We assess our model by comparing the predicted proportions of each phenotype over time with experimentally obtained phenotypic counts at 12, 20, 24, and 48-h growth. Predicted phenotypic proportions were obtained by varying values of *α*, *β*, *γ*, and the steepness of the sigmoid function in our model. We compared the cluster proportions obtained experimentally with model predictions. In the best-fit model, *U* remains as small clusters over *t* (*γ* = 0) and does not generate clusters of either size (Fig. 3C). The best-fit model (determined by the prediction error, see supplemental materials) accurately predicted the phenotypic proportions when compared with empirical distributions (Fig. 3D, E, *R*^2^ = 0.92).

We’ve previously shown using forward genetics that a recessive LOF mutation at the ACE2 locus is the genetic basis of the observed multicellular genotypes. Here, we confirm that the observed bistable system arises as a pleiotropic consequence of the LOF mutation in ACE2 that generates the selected multicellular phenotype. Through time-lapse microscopy, we directly observe the occurrence of phenotypic switching events, revealing some of the dynamics of the transitions between stages over time. Using mathematical modeling, we examine the phenotypic interrelationships to describe the phenotypic heterogeneity observed phenomena. Phenotypic distributions within the populations conform to a model in which the small clusters are produced by the large clusters and are the result of a transition between stages after the large clusters reach a threshold. Following a resource model, large clusters proliferate until a threshold at which small clusters start being produced. The threshold that triggers the transition between stages could be the result of multiple factors, including resource availability (i.e., glucose depletion), physical limitations related to size, or molecular stochastic processes. These results indicate that changes in the distribution of the two phenotypes in these populations can be modeled as the effect of both environmental and stochastic forces.

### Changes in expression profiles are associated with bistable phenotypes

**Fig. 4:**
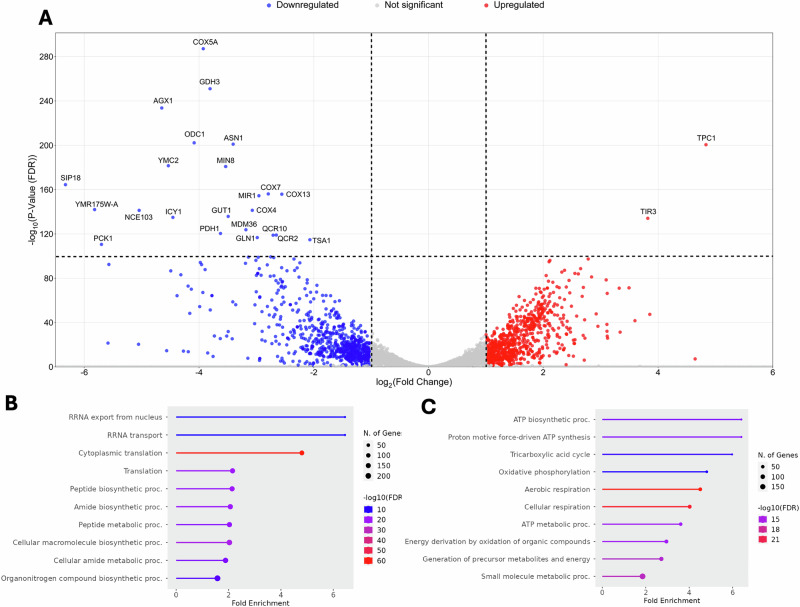
RNA-Seq expression profiles of the constructed *ace2*Δ *missense* isogenic strain Top vs. Bottom subpopulations. Top—smaller ancestral-like clusters—and Bottom—large multicellular clusters—phenotypic subpopulations present different expression profiles. **A** Volcano plot of −log2 fold-change (Log2FC) vs. log10 FDR-corrected *p* value (representing the probability of observed fold change under the null hypothesis of no expression difference) of Top/Bottom phenotypic subpopulations. The figure represents upregulated (red) and downregulated (blue) genes in the multicellular-enriched subpopulations. **B**, **C** GO enrichment analyses were performed using ShinyGO bioinformatics software. Graphs represent the Biological Process of the **B** upregulated and **C** downregulated Log2FC genes (for KEGG, GO cellular components, and GO Molecular function refer to Fig. S11). Fold enrichment indicates the ratio of genes belonging to a process compared to the background population of genes. Upregulated GO biological processes multicellular-enriched samples are associated with the ribosome, peptide, and sugar metabolism, and MAPK signaling. Downregulated processes are related to the mitochondrial membrane, aerobic respiration, and ATP metabolic processes.

We next sought to understand the molecular basis for the observed small and large clusters subpopulations underlying the bistable system. We mapped the observed morphotypes to differences in gene expression to gain insights into the cellular functions and biological processes. Comparing the expression profiles of the constructed *ace2Δ missense* isogenic strain subpopulations after gravitational selection, we found that 1939 genes (or 34.5% of all annotated genes) were differentially expressed (DE) between the Top—smaller ancestral-like clusters—and Bottom—large multicellular clusters—phenotypic subpopulations (*p* value < 0.05) of which 346 (6%) had more than a twofold difference in expression (Fig. 4A).

The magnitude of difference in gene expression profiles between the two size classes is not unlike what has been documented for cell cultures in different phases of growth in aerated environments, where significant metabolic shifts can happen between anaerobic fermentation of simple sugars to aerobic respiration of ethanol. Because individual cells from the different subpopulations likely experience differences in microenvironments and nutrient availability that might impact gene expression, we also compared gene expression patterns of the two phenotypes to those of WT cells taken from similar conditions, 24 h of growth in YPD (or post-diauxic growth phase), as well as 8 h of growth in YPD (late-logarithmic growth phase). In WT cultures, we found 47.5% of genes DE between cells in late logarithmic and post-diauxic growth phases. While there was considerable overlap in the gene set (1068 of 1939) found to be DE between the 8- vs. 24-h WT cultures and between the two *ace2Δ missense* subpopulations sampled at 24 h, the direction of differential expression was not consistent when comparing the two sets. This is evidenced by significant overlap when comparing genes DE in the *ace2Δ missense* Bottom (large clusters enriched) subcultures to genes DE between 24- and 8-h WT cultures, even when accounting for opposite LogFC values (−LogFC) (Fig. S9). A broader comparison of expression profiles among samples suggests that rather than mapping neatly onto any of the sampled WT culture states or even the undivided *ace2Δ missense* cultures sampled in the same conditions, the two subpopulations have distinct gene expression signatures with the large clusters-enriched Bottom subpopulations displaying physiological patterns more like log-phase samples than the small-clusters-enriched Top subpopulations (Fig. S10).

Expression level differences suggest that mitochondrial respiration and some pathways related to glutamate metabolism are downregulated in the Bottom subpopulations relative to Top subpopulations. Of the highly expressed genes in the Bottom large clusters-enriched samples (annotated in red in Fig. 4A), TPC1 is a member of the mitochondrial carrier family whose overexpression appears to increase invasiveness during filamentous growth, and TIR3 is expressed and required for anaerobic growth. Of the top 23 most downregulated genes (annotated in blue in Fig. 4A), 6 (COX4, COX5B, COX7A, COX13, QCR2, and QCR10) are involved in oxidative phosphorylation, 5 genes (AGX1, ASN1, GDH3, ODC1, and YMC2) linked to alanine, aspartate, and glutamate metabolism, and 5 genes (GLN1, GUT1, NCE103, PCK1 and PDH1) are associated with metabolic pathways. MDM36, MRA1, and MIR1 are mitochondrial proteins related to mitochondrial morphology, negatively regulate the respiratory complex assembly, and are involved in the import of inorganic phosphate, respectively. SIP18 is a phospholipid-binding hydrophilic protein essential for desiccation-rehydration. TAS1 codes for a peroxiredoxin, an antioxidant to protect against oxidative stress. YMR157W-A and ICY1 are proteins of unknown function; the latter is required for the viability of cells lacking mitochondrial DNA in rich media. There is a preponderance of expression level differences associated with mitochondrial function.

Using Gene Ontology (GO) enrichment analysis on gene sets, we identified DE genes of the two phenotypic subpopulations (Figs. 4B, C and S11). Upregulated genes in the Bottom large clusters-enriched subpopulations were related to the ribosome, peptide, and sugars metabolism, and mitogen-activated protein kinase (MAPK) signaling (Fig. 4B). GO biological process overrepresented categories are cellular metabolic processes, biological regulation, cellular component organization, ribosomal RNA transportation, and translation, related to the ribosome, cell membrane, and cell wall components, and correlated with RNA binding, ribosomal RNA binding, structural constituents of the ribosome and structural molecules activity. Downregulated genes in the Bottom subpopulations were significantly enriched with GO biological processes associated with aerobic respiration, cellular respiration, ATP biosynthesis, ATP metabolic processes, and generation of precursor metabolites and energy (Fig. 4C), mainly related to the mitochondrial membrane and linked to molecular functions related to molecule binding and catalytic activity.

Ace2p is responsible for controlling post-division separation of mother and daughter cells following mitosis via transcriptional control of a handful of proteins. Comparison of our *ace2Δ missense* cultures to WT cultures shows consistent downregulation of the 7 genes (DSE1, SCW11, AMN1, CTS1, SUN4, PRY3, and DSE4) tied to Ace2p transcriptional regulation. Of these, SCW11, PRY3, and SUN4 were divergently regulated in the Bottom compared to the Top subpopulations (see Fig. S12 for Bottom and Top subpopulations comparisons). Deviations from the expected pattern can be caused by compensatory regulation, context-dependent activation, or post-transcriptional effects. No systematic pattern of gene expression differences was observed for ACE2 and AIM44, previously identified as predominant causal loci in the evolution of multicellular clusters^37,38^. The diversity of expression levels among these snowflake multicellular loci reflects the complex mechanisms of cell division in *S. cerevisiae*^39^ and illuminates the complexity of cell size and gene expression previously observed in ACE2 heterozygotes^21^.

## Discussion

There is a long, contentious debate on rapid morphological evolution, particularly in non-stressful conditions, with strong expectations of modest, gradual responses to directional selection^5,40,41^. Here, we demonstrate the rapid evolution of genotypes with two phenotypic classes in response to non-stressful conditions. The same LOF mutation that gives rise to a derived phenotype consistent with a directional response to selection can also destabilize regulatory control, resulting in subpopulations of smaller individuals that resemble the ancestral state. Time-course assays and mathematical modeling indicate that the two phenotypic classes persist across tested conditions with temporally variable frequencies over the growth cycle, suggesting they are stable states rather than environmentally induced, and that the smaller ancestral-like phenotype arises directly from the growth of derived phenotypic individuals. Transcriptomic assays demonstrate that the two phenotypes are metabolically distinct, with individuals exhibiting the smaller ancestral-like phenotype showing quiescent gene expression. The dual outcome reflects how disruption of key regulatory loci not only shifts the mean phenotype but also amplifies stochastic variation at the division level. Thus, the maintenance of such mutations can be explained by the adaptive advantage of the derived phenotype, together with the pliability conferred through the continued presence of ancestral-like states. These evolutionary responses are consistent with stochastic regulatory control inherent in the unselected ancestral genetic background, illustrating the conditional basis for evolutionary responses to selection and opening avenues for further adaptation.

The *S. cerevisiae* experimentally evolved multicellular yeast system has been extensively studied to address questions regarding emergent multicellularity, reversibility, cost, and benefits of increased size, and development^20,22,42,43^. Taking a top-down approach (phenotype to genetic expression) uncovers phenotypic heterogeneity in the experimentally evolved multicellular system. Isogenic cells frequently exhibit heterogeneity in various phenotypic traits such as cell morphology, growth rate, gene expression, altered motility, or antibiotic resistance^44,45^. Phenotypic heterogeneity is typically regarded as an adaptive trait in microbial populations^23,24,46–48^, rather than a consequence of tradeoffs, pleiotropy, and ancestral plasticity. Bistability, in particular, is considered adaptive because it renders a dynamic system that may serve as a stabilizing strategy in the face of ecological shifts^49,50^. Instances of phenotypic heterogeneity that render bistability in isogenic populations include: division of labor^51^, bet-hedging^52^, negative frequency-dependent interactions^53^, group-level phenotypes and cellular vigor^47,54^. Given that our multicellular strains were obtained under directional selection for increased size over hundreds of generations, we do not consider the observed phenotypic heterogeneity a bet-hedging strategy but rather a pleiotropic consequence of a beneficial mutation for increased size via cluster formation. Complex phenotypic landscapes readily evolve through simple LOF mutations, opening up opportunities for evolutionary modification and adaptation. We note that unraveling phenotypic heterogeneity within genetically isogenic populations is complex, even in a model microbial system.

LOF mutations at the ACE2 locus are a phenotypic capacitor, even while generating a multicellular phenotype^15,42^. The bimodal distributions of the evolved multicellular genotype can be inferred from the population distribution average effects of the ACE2 mutations. LOF variation is considered a driver of adaptation and correlated with biodiversity^31,54,55^. LOF mutations have been observed in multiple experimental settings, preferentially targeting metabolic and regulatory pathways aiding adaptation to novel environments^56,57^. The adaptive relevance of LOF mutation is increasingly recognized in natural and clinical settings^54,55^. Our experimental approach reveals empirical evidence of phenotypic switching arising from LOF mutations in nascent multicellularity. It provides a tool to assess conceptual claims regarding the role of LOF mutations in the evolution of novelty^29^. Although the LOF mutation at the ACE2 locus is responsible for the bistable response at the cellular level, we observed that evolved strains present phenotypic differences between each other and when compared with the constructed strains. Previous work has shown differences in phenotypic expression of both derived strains^58,59^ despite coming from the same populations. This indicates an effect of the intra-genotype phenotypic variability, accumulated over the evolution of the multicellular strains. These results demonstrate that such observed phenotypic heterogeneity, whose quantitative parameters can be adjusted, is an evolvable trait, essential for subsequent adaptation via cellular differentiation as hypothesized.

The evolutionary process depends upon genetic variation, but transient phenotypes can provide an additional selectable layer of traits, increasing adaptive possibilities. Such transient variation is recognized as an evolutionary driving force in many biological systems^60,61^, but this is the first time experimentally addressing its role across a major evolutionary transition (previous theoretical work^12–14^). Here, we observed a steep decline in size when selecting for ancestral-like phenotypes. The repeatable response to selection in these isogenic strains indicates the adaptive potential of standing transient phenotypic variation. In line with our experimental findings, we hypothesize that phenotypic heterogeneity might provide alternative selectable pathways in the experimentally evolved multicellular yeast system that can be explored in the future. For example, one of the identified key problems in the transition towards multicellularity is stabilization, which limits reversion when selection tilts towards the ancestral state; unicellular growth^62^. Earlier research on experimentally evolved multicellularity has shown a fitness cost relative to the ancestral state in the absence of selective conditions^15,63^. Previous challenges in back-selecting for unicellularity from multicellular *S. cerevisiae* snowflake yeast are likely to stem from the observed bimodal phenotype^20^. Indeed, the exclusion of ancestral unicellular phenotypes by multicellular individuals during settling selection may have been accelerated by the phenotypic bimodality of the LOF mutation. This is challenging to investigate, given the concomitant phenotypic responses.

The process by which the system produces propagules of the small phenotype appears largely random, suggesting phenotypic switching is probably mediated by stochastic regulatory control, consistent with the persistent heterogeneity across conditions. However, we acknowledge that our data do not exclude the possibility that this phenomenon arises from a combination of plastic—in response to some unknown environmental variable that is heterogeneous in the yeast culture—and stochastic regulatory mechanisms. Gene expression, and its modulation by other genes or external factors, is necessarily stochastic, given the molecular basis for transcription and translation^64^. Observed phenotypic dynamics conform to a model in which small clusters are only produced by large multicellular clusters, because cells switch stochastically to a non-growing state, as confirmed via transcriptomics. Moreover, smaller ancestral-like clusters revert to the multicellular growth phenotype under favorable conditions, as we see experimentally. In our model, large clusters initially proliferate until a threshold triggers a transition between stages (smaller ancestral-like clusters). The threshold represents instances of yeast cell-to-cell heterogeneity, such as resource limitation (i.e., diffusion limitation within the large multicellular cluster^42^ or age-related oxidative burden resulting from oxidatively damaged proteins being preferentially retained in mother cells during division^65^). Therefore, we have support for cell-to-cell heterogeneity, environmentally or developmentally mediated, that triggers the proliferation of ancestral-like phenotypes. The stochastic phenotypic switching results from the unicellular ancestral asynchronous cell cycle behavior that increases cellular heterogeneity (see yeast stochastic cell cycle activation process^66–68^). Typically, this cellular stochastic phenotype switching is assumed to be adaptive in relatively stable environmental conditions with infrequent changes^69,70^. Examples of stochastic switching have been argued as an adaptation in bacterial persistence to antibiotics^71^, pathogenic bacteria^72^, in soil bacteria^73^, and quiescent yeast studies^74^. Similar to the above examples, an ancestral-like state can be regarded as a persister state (stationary phase) under environments in which there is a cost to growing cells. Our results demonstrate the ease with which stochastic phenotypic switching can evolve, even under directional selection to non-stressful conditions.

The capacity for phenotypic switching is a nearly universal feature in microorganisms^75^, resulting from transcriptomic and translational changes that alter gene expression^71,73^. Our model suggests large gene expression differences between the two phenotypes, with the ancestral-like individuals displaying a post-diauxic/stationary phase physiology. In the large clusters, the upregulation of metabolic pathways related to the ribosome and sugar metabolism suggests growth on fermentable sugars and the production of mRNAs for r-proteins, which is compatible with a log-phase proliferation state. These results support our model, in which large clusters divide actively, and smaller ancestral-like clusters are non-growing. Other non-developmental changes, like negative regulation of mitochondrial respiration, suggest changes in respiration in the large clusters despite their branched body plan. These changes are consistent with the developmental evolution observed by Bozdag et al.^76^, where fixed functional differences in respiration were observed in large clusters evolved under different O_2_ conditions. Some differences involved developmental pathways, i.e., the overexpression of the MAPK signaling pathways in the large clusters. MAPK cascade is a key signaling network that regulates the cell cycle, cell proliferation, and filamentous growth^77^, that can operate as a bistable switch^78^. Phenotypic heterogeneity at the gene expression level arises from ecological factors^79^, cell-inherent dynamics (cell division^80^ or aging^81^), and/or stochastic effects^82^. The link is remarkably complex and convoluted; results point to the MAPK cascade as a candidate network to further study; however, non-development changes in gene expression could arise from ecological factors and biological feedbacks. The observed metabolic differences and morphological heterogeneity appear mutually reinforcing, but do not allow us to establish causal direction—whether gene expression state or morphological outcome comes first. Moreover, it remains unclear from the transcriptomic results whether phenotypic switching is driven by ecological factors, intrinsic cellular dynamics, stochastic effects, or a combination thereof.

There are significant challenges in interpreting the evolutionary, genetic, and environmental causal basis for phenotypes. Genetic background and subtle environmental effects can substantially alter phenotypes, thereby calling into question any resulting selective benefits. Microbial experimental evolution studies are notable in reducing these challenges, but these difficulties are not completely eliminated^83,84^. In this study, LOF mutations in ACE2 are the causal genetic differences in generating the bimodal size distributions of small and large clusters. However, the causal biochemical and molecular mechanisms underlying the two distinct size classes remain uncertain. These mechanisms may involve inputs from intrinsic factors, such as stochastic gene expression, extrinsic factors, such as microenvironmental modulation, or some combination of both. The transcriptional profiles of the two size classes suggest that gene expression shifts during cell replication may be involved, consistent with the role of the ACE2 locus in regulating cell division. The surprising consequence of a bimodal evolutionary response to direction selection supports further work in disentangling these mechanisms.

We capitalize on experimentally evolved multicellularity in *S. cerevisiae* to investigate the phenotypic consequences of directional selection for increased size. Demonstrating that LOF mutations in both constructed and evolved strains, while leading to the emergence of multicellular clusters, concomitantly generate smaller ancestral-like clusters that are metabolically distinct. Time-course assays and mathematical modeling indicate that smaller clusters arise directly from the growth of derived phenotypic individuals. Experimental data and transcriptional profiles are not readily explained by microenvironmental differences, suggesting that the observed regulatory destabilization and maintenance of bistability across diverse conditions are more consistent with a stochastic mechanism than a classical plastic response to an unknown cue. Phenotypic heterogeneity among genotypes with similar or identical LOF mutations substantiates that phenotypic switching can be an evolvable trait. Altogether, this is a striking example of how phenotypic heterogeneity can act as an evolutionary forerunner, maintaining biological diversity without compromising genetic stability in the initial stages of this transition. We hypothesize that phenotypic heterogeneity is one of the building blocks for evolutionary novelty, offering potential states upon which selection can act, and facilitating the emergence of novel multicellular forms.

## Methods

### Study system, constructed strains, and culture conditions

All strains studied were constructed or derived from clonal Y55 strains, a diploid wild-type (WT) *S. cerevisiae* yeast. Strains C1W8.1 and C1W8.2 are multicellular-derived genotypes of Y55, isolated from the same population after 8 weeks of settling selection in YPD media (refer to Ratcliff et al.^15^).

The ACE2 knockout (Y55 ace2∆::kanMX) and ACE2 missense (Y55 ace2∆::kanMX+ace2c.^1934A>T^) strains, hereon referred as *ace2Δ knockout* and *ace2Δ missense*, were generated via replacement of the ACE2 locus in isogenic WT Y55 isolates using the LiOAc/PEG transformation protocol^85^ To generate the replacement cassettes, we used overlap-extension PCR (for primers see Table S6). The kanMX gene was derived from the plasmid pFA6-kanMX (Addgene ID plasmid #39296). The kanMX+ace2c.^1934A>T^ cassette was made by adding kanMX downstream of the ACE2 locus in the experimentally evolved multicellular strain C1W8.l. Transformants were selected by replica plating on YPD with G418 bisulfate (100 μg/ml; Thermo Fisher).

The multicellularity-generating effects of ACE2 are largely recessive^21^. We generated homozygous strains for the desired cassette by sporulating transformant heterozygous strains in 10 ml of KAc medium (2% potassium acetate, 0.5 g dextrose). Haploid spores were separated using a tetrad dissection microscope Kit-C accessory (Micro Video Instruments Inc.) mounted in the mechanical stage of a Nikon optic microscope (Nikon TE2000) and allowed to auto-diploidize on YPD. Homozygous colonies were selected based on colony morphology and growth on YPD plates with G418 bisulfate (100 μg/ml; Thermo Fisher). Multicellular strains form colonies with a rugose edge and wrinkled surface (hereon referred to as rugose) in contrast to WT *S. cerevisiae* smooth colonies (Fig. S13). All transformants were validated using PCR and gel electrophoresis. Additional validation of the Y55 ace2∆::kanMX+ace2c.^1934A>T^ was done via variant calling for one of the RNA-seq samples (top1 adhesion number SRR32105384, see below) using STAR^86^ alignments to the Y55 reference assembly (GCA_903819135.2), the GATK^87^ best practices pipeline (https://gatk.broadinstitute.org/hc/en-us/articles/360035531192-RNAseq-short-variant-discovery-SNPs-Indels), and snpEff^87^ (see Variant call format file in ref. ^88^).

Strains were grown in 25 × 150 mm glass culture tubes with 10 ml of media at 30°C and shaking at 250 rpm. The growth media used in this study were Yeast Peptone Dextrose media (YPD; 1% (v/w) yeast extract, 2% (v/w) peptone, 2% (v/w) D-glucose, pH 5.8, 1.5% (v/w) agar for solid media). For the selection of resistance cells, G418 bisulfate (Thermo Fisher) was added to YPD plates at a final concentration of 100 μg/ml.

### Microscopy phenotypic assays

Individual particles within derived and constructed genotypes were classified based on size phenotypes in two distinct morphotypes: (i) multicellular large clusters, referring to a group of cells that remained together after divisions, comprising multiple cells displaying a range of sizes, and (ii) small ancestral-like clusters, those phenotypes consisting of one to a few cells. Phenotypes of the WT Y55 ancestor strain are referred to as unicellular.

Microphotographs were acquired using a Nikon TE2000 microscope equipped with 10x and 20x objectives. Images were processed with ImageJ^89^ and FIJI^90^ software. Contrast was first enhanced by 35% using the “Enhance Contrast” function, followed by edge detection with the “Find Edges” command. Images were then converted to binary format by applying a threshold to separate particles from the brighter background. Binary masks were refined by applying the “Fill Holes” command, and adjacent particles were separated using the “Watershed” algorithm. Particle features were quantified using the “Analyze Particles” function. Particles were classified into two categories—multicellular large clusters and ancestral-like small phenotypes—based on their area (in squared pixels). For 10× images, a size threshold of 7000 squared pixels was used as a cutoff point for classification, corresponding to the maximum size observed in the unicellular ancestor.

Time-lapse videos were obtained with a Nikon TE2000 microscope using a 10x objective. Images were captured at fixed intervals of 3, 5, or 15 min up to 26 h, using the time-lapse acquisition setting of the NIS-Elements D software. Isogenic cultures of the C1W8.1-derived, *ace2Δ knockout* and *ace2Δ missense* strains grew for 24 h in YPD medium. Approximately 200 µL of each overnight culture was transferred into a FlowCell FC50 (Fluid Imaging Technologies, Inc.) pre-filled with YPD medium. The FlowCell was clamped at both ends to minimize evaporation. Time-lapse timestamps were added with ImageJ^89^ and FIJI^90^ software.

### Populations particle size distributions

Population particle size distributions were measured by an electronic particle counter (Multisizer 4 Coulter Counter® (Beckman Coulter)) and a digital flow cytometer (FlowCam® 3.0 Fluid Imaging Technologies). The Coulter Counter® particle counter was calibrated with 5 µm diameter standard calibration beads (Beckman Coulter) before every analysis. PBS solution (phosphate-buffered saline) was used as the system’s electrolyte. Samples of 5 µL from isogenic populations grown at different time intervals (12 h, 20 h, 24 h, and 48 h) were added to the instrument’s measuring beaker filled with 10 ml PBS. Consecutive measurements of 300 µL of the dissolution were recorded using a 70 mm aperture tube. The aperture tube was flushed with PBS between samples. Particle diameter (µm) is estimated as the equivalent spherical diameter. Raw Multisizer files (*.#m4) were batch-processed with the R package MultisizerToolkit (https://github.com/gettl008/MultisizerToolkit).

Flow cytometer micrographs were obtained in a FlowCam 3.0 using 10× (multicellular genotypes) and ×20 (WT genotype) objective lenses and FC100 and FC50 FlowCells (Fluid Imaging Technologies, Inc.), respectively. The instrument was calibrated using 5, 10, and 20 µm size beads (Fluid Imaging Technologies, Inc.). The thresholds in particle diameter are >3 μm (20× objective lens) and >5 μm (10× objective lens). An initial fixed number of particles (*N* = 5000) were imaged per sample at a flow rate of 0.07 ml/min. The Area Base Diameter is calculated from the number of gray-scale pixels of the binary image and converted into a circle with the same number of pixels.

### Single colony assays

The small ancestral-like clusters subgroup of the evolved and constructed strains was streaked on YPD plates and grown for 48 h at 30 °C. The small clusters subgroup of the populations was obtained by taking the top 100 μL aliquots from a 1.5 mL overnight-grown culture after 7 min of benchtop settling. After 7 min, most of the population settles and forms a pellet at the bottom of the centrifuge tube. Individual rugose (Fig. S13) colonies were randomly picked and resuspended in 500 µL of YPD. Initial genotypes and resuspended randomly selected colonies were measured with an electronic particle counter (Multisizer 4 Coulter Counter® (Beckman Coulter)).

Six colony lineages (CL) (three of each for evolved strain, C1W8.1 and C1W8.2) were maintained over three cycles of selection by benchtop settling. A cycle of selection consisted of randomly selecting a single rugose colony, resuspending it in 500 µL of YPD, transferring 100 µL of the resuspended aliquot into liquid YPD for overnight growth, and then plating the fraction containing small clusters after benchtop setting. Resuspended colonies were measured with an electronic particle counter (Multisizer 4 Coulter Counter® (Beckman Coulter)).

### Controlled experimental conditions setup

Ancestral, derived, and constructed genotypes were cultured under different controlled experimental conditions. Single colonies per strain were picked and grown overnight in glass culture tubes with 10 ml of YPD media at 30 °C and shaking at 250 rpm. Aliquots of each population were transferred into three different culturing conditions using a 1/100 dilution factor. Cultures were grown in: (i) 25 × 150 mm glass culture tubes with 10 ml of media, (ii) 50 ml capacity Erlenmeyer flasks (Pyrex narrow mouth, 78 mm height) with 10 ml, and (iii) 50 ml capacity Erlenmeyer flasks (Pyrex narrow mouth, 78 mm height) with 30 ml of media above the 20% capacity to reduce oxygen transfer. The three sets of culturing conditions were carried out with no shaking or with shaking at 250 rpm on a platform shaker (Innova 2000). YPD media was used across all conditions. All cultures were incubated in the same incubator for 24 h at 30 °C. Samples were taken from each condition and carefully mixed with a pipette to be assessed under optical microscopy. Microphotographs of each condition and strain were obtained with a Nikon TE2000 microscope using a 10× objective.

### DNA and RNA sequencing

Genomic DNA was extracted from isogenic isolates of C1W8.1 to generate the replacement cassettes to construct *ace2Δ missense* strains (Y55 ace2∆:kanMX+ace2c.^1934A>T^, for primers see Table S6). DNA extraction was performed using the zymolyase enzymatic purification method (see Biolabs Genomic DNA Extraction from Yeast (NEB #T3010)). PCR thermocycler protocol to amplify the replacement cassettes consisted of denaturation at 98 °C for 1–3 min, followed by 32 cycles of denaturation at 98 °C for 10 s, annealing at 68 °C for 30 s, and extension at 72 °C for 2 min, followed by a final extension at 72 °C for 10 min. PCR reaction success was evaluated via agarose gel electrophoresis.

To determine the molecular mechanistic underpinnings of each of the observed phenotypic size classes (non-settling small clusters and settling large clusters), RNA was extracted from a constructed *ace2Δ missense* strain using an Invitrogen® PureLink RNA Mini Kit. Four biological replicates from an isogenic *ace2Δ missense* strain were grown for 24 h in shaken batch cultures of 10 ml YPD at 30 °C. Aliquots of the same population were taken from the Top and Bottom of the tube after 7 min of bench settling without disruption. The three extracted samples, out of four, per treatment with the highest RNA integrity score were submitted for TrueSeq Stranded RNA-Seq. TrueSeq Stranded RNA-Seq, NextSeq2000 P2 100 cycles, 50 paired-end sequencing technology was performed by the University of Minnesota Genomics Center. The final RNA-seq output range is ~75–78 million paired-end reads for the top samples, and ~79–89 million paired-end reads for the Bottom samples.

As a control and to assess how microenvironmental differences between morphotypes might influence differences in transcriptional profiles, we also sequenced total RNA from wild-type Y55 and unseparated *ace2Δ missense* cultures extracted at two phases of population growth in oxygenated (shaking) YPD: late log-phase (8 h of growth in YPD) and post-diauxic phase (24 h of growth in YPD). These two growth phases reflect distinctly different carbon-availability environments: dextrose availability and dextrose depletion/ethanol availability, respectively. For these samples, RNA was extracted from three replicates at each phase using the Qiagen RNeasy Mini Kit, and stranded TruSeq mRNA libraries were sequenced on an Illumina NovaSeq.

### Statistics and reproducibility

Statistical analysis was performed using R^91^ and JMP Pro 16 (SAS Institute Inc). Diameter (μm) frequency distributions are described using standard descriptive statistics. The non-Gaussian multimodal distributions for multicellular strains were examined using two-component normal mixture models to extract individual peak information (“mixtools”/“normalmix” and “mclust” in R). The overlapping index (*η*) of the diameter frequency distributions was computed using the R-package “overlapping” ^92^. Hierarchical clustering of η was performed with Euclidean distances using the Ward D2 method with the “hclust” R-package.

Raw RNA-seq reads were quantified with Salmon (v1.2.1)^93^ and mapped to gene sequences derived from the Y55 assembly (GCA_903819135.2). DE genes were determined using edgeR (v3.2.1)^94^. DE genes were based on pairwise exact tests and filtered for BH-adjusted *p* values less than 0.05. Similarity among sample replicates was evaluated using multidimensional scaling of expression values using the Multidimensional Scaling plot function in limma^95^ and dispersion estimates from edgeR. Comparisons of sets of DE genes were evaluated using rank-rank hypergeometric overlap tests in R^92^, where genes were ranked by signed log10 BH-adjusted *p* values. Volcano plots and bar charts were generated using the matplotlib^96^ and seaborn libraries^97^.

GO enrichment analyses were performed with ShinyGo 0.8^98^. Our gene list consisted of those genes upregulated or downregulated more than twofold compared to the background population of ~6100 genes. False discovery rate (FDR) adjusted *P* values (cutoff *p* < 0.05) are calculated using the Benjamini–Hochberg method to control type I errors.

### Reporting summary

Further information on research design is available in the Nature Portfolio Reporting Summary linked to this article.

## Supplementary information

Supplemental Material

Description of Additional Supplementary Materials

Supplementary Movie 1

Supplementary Movie 2

Supplementary Movie 3

Reporting Summary

## Data Availability

All data described or analyzed in this study are included in the main text, SI Appendix, and raw data. Raw data can be found in the Zenodo repository 10.5281/zenodo.15652984. RNA-seq raw sequences will be released under the NCBI-GenBank. BioProject PRJNA1214970 accession number after publication. Genome reference *Saccharomyces cerevisiae* Y55 used in the analysis can be found under GCA_903819135.2 accession number and BioSample SAMEA6932012.

## References

[1] van’t Hof, A. E. et al. The industrial melanism mutation in British peppered moths is a transposable element. *Nature* **534**, 102–105 (2016). 27251284 10.1038/nature17951

[2] Lamichhaney, S. et al. A beak size locus in Darwin’s finches facilitated character displacement during a drought. *Science* **352**, 470–474 (2016). 27102486 10.1126/science.aad8786

[3] Baquero, F. et al. Evolutionary pathways and trajectories in antibiotic resistance. *Clin. Microbiol. Rev.* **34**, e0005019 (2021). 34190572 10.1128/CMR.00050-19 PMC8404696

[4] Orr, H. A. The population genetics of adaptation: the distribution of factors fixed during adaptive evolution. *Evolution* **52**, 935–949 (1998). 28565213 10.1111/j.1558-5646.1998.tb01823.x

[5] Fisher, R. A. *The Genetical Theory of Natural Selection* Vol. xiv, 272. 10.5962/bhl.title.27468 (Clarendon Press, 1930).

[6] Lou, J. et al. Predictive evolutionary modelling for influenza virus by site-based dynamics of mutations. *Nat. Commun.* **15**, 2546 (2024). 38514647 10.1038/s41467-024-46918-0 PMC10958014

[7] Denison, R. F. Frontmatter. in *Darwinian Agriculture: How Understanding Evolution Can Improve Agriculture* i–iv (Princeton University Press, 2012) https://press.princeton.edu/books/paperback/9780691173764/darwinian-agriculture?srsltid=AfmBOoqdJcJk5qx3QHRvibJaQjkRBEINs42fljX1ZIzXfDo70Tq8MhW.

[8] Turcotte, M. M., Corrin, M. S. C. & Johnson, M. T. J. Adaptive evolution in ecological communities. *PLOS Biol.* **10**, e1001332 (2012). 22615542 10.1371/journal.pbio.1001332 PMC3352851

[9] Shaw, R. G. From the past to the future: considering the value and limits of evolutionary prediction. * Am. Nat.* **193**, 1–10 (2019). 30624100 10.1086/700565

[10] Bull, J. J. & Antia, R. Which ‘imperfect vaccines’ encourage the evolution of higher virulence? *Evol. Med. Public Health* **10**, 202–213 (2022). 35539897 10.1093/emph/eoac015 PMC9081871

[11] Grosberg, R. K. & Strathmann, R. R. The evolution of multicellularity: a minor major transition? *Annu. Rev. Ecol. Evol. Syst.* **38**, 621–654 (2007).

[12] Davison, D. R., Nedelcu, A. M., Eneji, O. D. A. & Michod, R. E. Plasticity and the evolution of group-level regulation of cellular differentiation in the volvocine algae. *Proc. R. Soc. B Biol. Sci.* **292**, 20242477 (2025). 10.1098/rspb.2024.2477 PMC11920831 40103550

[13] Moczek, A. P. et al. The role of developmental plasticity in evolutionary innovation. *Proc. Biol. Sci.* **278**, 2705–2713 (2011). 21676977 10.1098/rspb.2011.0971 PMC3145196

[14] Sebé-Pedrós, A., Degnan, B. M. & Ruiz-Trillo, I. The origin of Metazoa: a unicellular perspective. *Nat. Rev. Genet.* **18**, 498–512 (2017). 28479598 10.1038/nrg.2017.21

[15] Ratcliff, W. C., Denison, R. F., Borrello, M. & Travisano, M. Experimental evolution of multicellularity. *Proc. Natl. Acad. Sci. USA* **109**, 1595–1600 (2012). 22307617 10.1073/pnas.1115323109 PMC3277146

[16] Driscoll, W. W. & Travisano, M. Synergistic cooperation promotes multicellular performance and unicellular free-rider persistence. *Nat. Commun.* **8**, 15707 (2017). 28580966 10.1038/ncomms15707 PMC5465372

[17] Baselga-Cervera, B., Jacobsen, K. A., Ford Denison, R. & Travisano, M. Experimental evolution in the cyanobacterium Trichormus variabilis: increases in size and morphological diversity. *Evolution* **77**, 1216–1225 (2023). 36821408 10.1093/evolut/qpad037

[18] Herron, M. D. et al. De novo origins of multicellularity in response to predation. *Sci. Rep.* **9**, 2328 (2019). 30787483 10.1038/s41598-019-39558-8 PMC6382799

[19] Conlin, P. L. Microbial evolution in changing environments.Thesis (Ph.D.) (University of Washington, 2018).

[20] Rebolleda-Gómez, M. & Travisano, M. Adaptation, chance, and history in experimental evolution reversals to unicellularity. *Evolution* **73**, 73–83 (2019). 30520011 10.1111/evo.13654 PMC6590667

[21] Baselga-Cervera, B., Gettle, N. & Travisano, M. Loss-of-heterozygosity facilitates a fitness valley crossing in experimentally evolved multicellular yeast. *Proc. R. Soc. B Biol. Sci.* **289**, 20212722 (2022). 10.1098/rspb.2021.2722 PMC9185828 36547392

[22] Ratcliff, W. C., Fankhauser, J. D., Rogers, D. W., Greig, D. & Travisano, M. Origins of multicellular evolvability in snowflake yeast. *Nat. Commun.* **6**, 6102 (2015). 25600558 10.1038/ncomms7102 PMC4309424

[23] Avery, S. V. Microbial cell individuality and the underlying sources of heterogeneity. *Nat. Rev. Microbiol* **4**, 577–587 (2006). 16845428 10.1038/nrmicro1460

[24] Govers, S. K., Adam, A., Blockeel, H. & Aertsen, A. Rapid phenotypic individualization of bacterial sister cells. *Sci. Rep.* **7**, 8473 (2017). 28814770 10.1038/s41598-017-08660-0 PMC5559607

[25] Fridman, O., Goldberg, A., Ronin, I., Shoresh, N. & Balaban, N. Q. Optimization of lag time underlies antibiotic tolerance in evolved bacterial populations. *Nature* **513**, 418–421 (2014). 25043002 10.1038/nature13469

[26] Siebring, J. et al. Repeated triggering of sporulation in Bacillus subtilis selects against a protein that affects the timing of cell division. *ISME J.* **8**, 77–87 (2014). 23924781 10.1038/ismej.2013.128 PMC3869009

[27] Raj, A. & van Oudenaarden, A. Stochastic gene expression and its consequences. *Cell* **135**, 216–226 (2008). 18957198 10.1016/j.cell.2008.09.050 PMC3118044

[28] Schröter, L. & Dersch, P. Phenotypic diversification of microbial pathogens—cooperating and preparing for the future. *J. Mol. Biol.* **431**, 4645–4655 (2019). 31260693 10.1016/j.jmb.2019.06.024

[29] Murray, A. W. Can gene-inactivating mutations lead to evolutionary novelty? *Curr. Biol.* **30**, R465–R471 (2020). 32428483 10.1016/j.cub.2020.03.072

[30] Albalat, R. & Cañestro, C. Evolution by gene loss. *Nat. Rev. Genet* **17**, 379–391 (2016). 27087500 10.1038/nrg.2016.39

[31] Monroe, J. G., McKay, J. K., Weigel, D. & Flood, P. J. The population genomics of adaptive loss of function. *Heredity* **126**, 383–395 (2021). 33574599 10.1038/s41437-021-00403-2 PMC7878030

[32] King, M. C. & Wilson, A. C. Evolution at two levels in humans and chimpanzees. *Science* **188**, 107–116 (1975). 1090005 10.1126/science.1090005

[33] McGregor, A. P. et al. Morphological evolution through multiple cis-regulatory mutations at a single gene. *Nature* **448**, 587–590 (2007). 17632547 10.1038/nature05988

[34] Scarcelli, N., Cheverud, J. M., Schaal, B. A. & Kover, P. X. Antagonistic pleiotropic effects reduce the potential adaptive value of the FRIGIDA locus. *Proc. Natl. Acad. Sci. USA* **104**, 16986–16991 (2007). 17940010 10.1073/pnas.0708209104 PMC2040464

[35] Tadrowski, A. C., Evans, M. R. & Waclaw, B. Phenotypic switching can speed up microbial evolution. *Sci. Rep.* **8**, 8941 (2018). 29895935 10.1038/s41598-018-27095-9 PMC5997679

[36] Stajic, D., Bank, C. & Gordo, I. Adaptive potential of epigenetic switching during adaptation to fluctuating environments. *Genome Biol. Evol.* **14**, evac065 (2022). 35567483 10.1093/gbe/evac065 PMC9113428

[37] Gettle, N. Causes and consequences of evolutionary innovation: an experimental approach to evaluating assumptions and predictions in macroevolutionary theory. (2020).

[38] Wang, P., Driscoll, W. W. & Travisano, M. Genomic sequencing reveals convergent adaptation during experimental evolution in two budding yeast species. *Commun. Biol.* **7**, 1–9 (2024). 38971878 10.1038/s42003-024-06485-y PMC11227552

[39] Münzner, U., Klipp, E. & Krantz, M. A comprehensive, mechanistically detailed, and executable model of the cell division cycle in Saccharomyces cerevisiae. *Nat. Commun.* **10**, 1308 (2019). 30899000 10.1038/s41467-019-08903-w PMC6428898

[40] Kingsolver, J. G. & Diamond, S. E. Phenotypic selection in natural populations: what limits directional selection? *Am. Nat.* **177**, 346–357 (2011). 21460543 10.1086/658341

[41] Katsnelson, M. I., Wolf, Y. I. & Koonin, E. V. On the feasibility of saltational evolution. *Proc. Natl. Acad. Sci. USA* **116**, 21068–21075 (2019). 31570621 10.1073/pnas.1909031116 PMC6800335

[42] Ratcliff, W. C. & Travisano, M. Experimental evolution of multicellular complexity in Saccharomyces cerevisiae. *Bioscience* **64**, 383–393 (2014).

[43] Rebolleda-Gómez, M. & Travisano, M. The cost of being big: local competition, importance of dispersal, and experimental evolution of reversal to unicellularity. * Am. Nat.* **192**, 731–744 (2018). 30444659 10.1086/700095

[44] Davidson, C. J. & Surette, M. G. Individuality in bacteria. *Annu. Rev. Genet.* **42**, 253–268 (2008). 18652543 10.1146/annurev.genet.42.110807.091601

[45] Holland, S. L., Reader, T., Dyer, P. S. & Avery, S. V. Phenotypic heterogeneity is a selected trait in natural yeast populations subject to environmental stress. *Environ. Microbiol.* **16**, 1729–1740 (2014). 24000788 10.1111/1462-2920.12243 PMC4231229

[46] Kümmerli, R. & Frank, S. A. Evolutionary explanations for heterogeneous behavior in clonal bacterial populations. *Trends Microbiol.* **31**, 665–667 (2023). 37117073 10.1016/j.tim.2023.04.003

[47] Magdanova, L. A. & Golyasnaya, N. V. Heterogeneity as an adaptive trait of microbial populations. *Microbiology* **82**, 1–10 (2013). 10.7868/s0026365613010072 23718043

[48] Balaban, N. Q., Merrin, J., Chait, R., Kowalik, L. & Leibler, S. Bacterial persistence as a phenotypic switch. *Science* **305**, 1622–1625 (2004). 15308767 10.1126/science.1099390

[49] Visco, P., Allen, R. J., Majumdar, S. N. & Evans, M. R. Switching and growth for microbial populations in catastrophic responsive environments. *Biophys. J.* **98**, 1099–1108 (2010). 20371309 10.1016/j.bpj.2009.11.049 PMC2849059

[50] Yao, Z., Davis, R. M., Kishony, R., Kahne, D. & Ruiz, N. Regulation of cell size in response to nutrient availability by fatty acid biosynthesis in Escherichia coli. *Proc. Natl. Acad. Sci. USA* **109**, E2561–E2568 (2012). 22908292 10.1073/pnas.1209742109 PMC3458391

[51] Beaumont, H. J. E., Gallie, J., Kost, C., Ferguson, G. C. & Rainey, P. B. Experimental evolution of bet hedging. *Nature* **462**, 90–93 (2009). 19890329 10.1038/nature08504

[52] Healey, D., Axelrod, K. & Gore, J. Negative frequency-dependent interactions can underlie phenotypic heterogeneity in a clonal microbial population. *Mol. Syst. Biol.* **12**, 877 (2016). 27487817 10.15252/msb.20167033 PMC5119493

[53] Ackermann, M. A functional perspective on phenotypic heterogeneity in microorganisms. *Nat. Rev. Microbiol.* **13**, 497–508 (2015). 26145732 10.1038/nrmicro3491

[54] Xu, Y.-C. & Guo, Y.-L. Less is more, natural loss-of-function mutation is a strategy for adaptation. *Plant Commun.* **1**, 100103 (2020). 33367264 10.1016/j.xplc.2020.100103 PMC7743898

[55] Helsen, J. et al. Gene loss predictably drives evolutionary adaptation. *Mol. Biol. Evol.* **37**, 2989–3002 (2020). 32658971 10.1093/molbev/msaa172 PMC7530610

[56] Hottes, A. K. et al. Bacterial adaptation through loss of function. *PLoS Genet.* **9**, e1003617 (2013). 23874220 10.1371/journal.pgen.1003617 PMC3708842

[57] Klim, J., Zielenkiewicz, U. & Kaczanowski, S. Loss-of-function mutations are main drivers of adaptations during short-term evolution. *Sci. Rep.* **14**, 7128 (2024). 38532077 10.1038/s41598-024-57694-8 PMC10965932

[58] Rebolleda-Gómez, M., Ratcliff, W. C., Fankhauser, J. & Travisano, M. Evolution of simple multicellularity increases environmental complexity. Preprint at 10.1101/067991 (2016).

[59] Rebolleda-Gomez, M., Ratcliff, W. & Travisano, M. Adaptation and divergence during experimental evolution of multicellular Saccharomyces cerevisiae. In *Proc. ALIFE 2012: The Thirteenth International Conference on the Synthesis and Simulation of Living Systems* 99–104. 10.1162/978-0-262-31050-5-ch014 (MIT Press Direct, 2012).

[60] Acar, M., Mettetal, J. T. & van Oudenaarden, A. Stochastic switching as a survival strategy in fluctuating environments. *Nat. Genet.* **40**, 471–475 (2008). 18362885 10.1038/ng.110

[61] Carja, O. & Plotkin, J. B. The evolutionary advantage of heritable phenotypic heterogeneity. *Sci. Rep.* **7**, 5090 (2017). 28698577 10.1038/s41598-017-05214-2 PMC5505965

[62] Libby, E., Ratcliff, W., Travisano, M. & Kerr, B. Geometry shapes evolution of early multicellularity. *PLoS Comput. Biol.* **10**, e1003803 (2014). 25233196 10.1371/journal.pcbi.1003803 PMC4168977

[63] Rainey, P. B. & Rainey, K. Evolution of cooperation and conflict in experimental bacterial populations. *Nature* **425**, 72–74 (2003). 12955142 10.1038/nature01906

[64] Swain, P. S., Elowitz, M. B. & Siggia, E. D. Intrinsic and extrinsic contributions to stochasticity in gene expression. *Proc. Natl. Acad. Sci. USA* **99**, 12795–12800 (2002). 12237400 10.1073/pnas.162041399 PMC130539

[65] Aguilaniu, H., Gustafsson, L., Rigoulet, M. & Nyström, T. Asymmetric inheritance of oxidatively damaged proteins during cytokinesis. *Science* **299**, 1751–1753 (2003). 12610228 10.1126/science.1080418

[66] Gao, X., Zhou, P. & Li, F. The multiple activations in budding yeast S-phase checkpoint are Poisson processes. *PNAS Nexus* **2**, pgad342 (2023). 37941810 10.1093/pnasnexus/pgad342 PMC10629469

[67] Perrino, G. et al. Automatic synchronisation of the cell cycle in budding yeast through closed-loop feedback control. *Nat. Commun.* **12**, 2452 (2021). 33907191 10.1038/s41467-021-22689-w PMC8079375

[68] Talia, S. D., Skotheim, J. M., Bean, J. M., Siggia, E. D. & Cross, F. R. The effects of molecular noise and size control on variability in the budding yeast cell cycle. *Nature* **448**, 947–951 (2007). 17713537 10.1038/nature06072

[69] Kussell, E. & Leibler, S. Phenotypic diversity, population growth, and information in fluctuating environments. *Science* **309**, 2075–2078 (2005). 16123265 10.1126/science.1114383

[70] Kussell, E., Kishony, R., Balaban, N. Q. & Leibler, S. Bacterial persistence. *Genetics* **169**, 1807–1814 (2005). 15687275 10.1534/genetics.104.035352 PMC1449587

[71] Kærn, M., Elston, T. C., Blake, W. J. & Collins, J. J. Stochasticity in gene expression: from theories to phenotypes. *Nat. Rev. Genet.* **6**, 451–464 (2005). 15883588 10.1038/nrg1615

[72] Moxon, R. & Kussell, E. The impact of bottlenecks on microbial survival, adaptation, and phenotypic switching in host–pathogen interactions. *Evolution* **71**, 2803–2816 (2017). 28983912 10.1111/evo.13370 PMC5722657

[73] Maamar, H., Raj, A. & Dubnau, D. Noise in gene expression determines cell fate in Bacillus subtilis. *Science* **317**, 526–529 (2007). 17569828 10.1126/science.1140818 PMC3828679

[74] Kumar, R. & Srivastava, S. Quantitative proteomic comparison of stationary/G0 phase cells and tetrads in budding yeast. *Sci. Rep.* **6**, 32031 (2016). 27558777 10.1038/srep32031 PMC4997312

[75] Rainey, P. B. et al. The evolutionary emergence of stochastic phenotype switching in bacteria. *Microb. Cell Factories* **10**, S14 (2011). 10.1186/1475-2859-10-S1-S14 PMC3231921 21995592

[76] Bozdag, G. O. et al. De novo evolution of macroscopic multicellularity. *Nature* 1–8 10.1038/s41586-023-06052-1 (2023). 10.1038/s41586-023-06052-1 PMC10425966 37165189

[77] Granek, J. A. & Magwene, P. M. Environmental and genetic determinants of colony morphology in yeast. *PLOS Genet.* **6**, e1000823 (2010). 20107600 10.1371/journal.pgen.1000823 PMC2809765

[78] Markevich, N. I., Hoek, J. B. & Kholodenko, B. N. Signaling switches and bistability arising from multisite phosphorylation in protein kinase cascades. *J. Cell Biol.* **164**, 353–359 (2004). 14744999 10.1083/jcb.200308060 PMC2172246

[79] Campbell, K., Herrera-Dominguez, L., Correia-Melo, C., Zelezniak, A. & Ralser, M. Biochemical principles enabling metabolic cooperativity and phenotypic heterogeneity at the single cell level. *Curr. Opin. Syst. Biol.* **8**, 97–108 (2018).

[80] Papagiannakis, A., Niebel, B., Wit, E. C. & Heinemann, M. Autonomous metabolic oscillations robustly gate the early and late cell cycle. *Mol. Cell* **65**, 285–295 (2017). 27989441 10.1016/j.molcel.2016.11.018

[81] Janssens, G. E. et al. Protein biogenesis machinery is a driver of replicative aging in yeast. *eLife* **4**, e08527 (2015). 26422514 10.7554/eLife.08527 PMC4718733

[82] Raj, A. & van Oudenaarden, A. Nature, nurture, or chance: stochastic gene expression and its consequences. *Cell* **135**, 216–226 (2008). 18957198 10.1016/j.cell.2008.09.050 PMC3118044

[83] Lenski, R. E. & Mittler, J. E. The directed mutation controversy and neo-Darwinism. *Science* **259**, 188–194 (1993). 7678468 10.1126/science.7678468

[84] Pribis, J. P., Zhai, Y., Hastings, P. J. & Rosenberg, S. M. Stress-induced mutagenesis, gambler cells, and stealth targeting antibiotic-induced evolution. *mBio* **13**, e01074–22 (2022). 35658528 10.1128/mbio.01074-22 PMC9239211

[85] Gardner, J. M. & Jaspersen, S. L. Manipulating the Yeast Genome: Deletion, Mutation, and Tagging by PCR. in *Yeast Genetics: Methods and Protocols* (eds Smith, J. S. & Burke, D. J.) 45–78. 10.1007/978-1-4939-1363-3_5 (Springer, 2014). 10.1007/978-1-4939-1363-3_5 25213239

[86] Dobin, A. et al. STAR: ultrafast universal RNA-seq aligner. *Bioinformatics* **29**, 15–21 (2013). 23104886 10.1093/bioinformatics/bts635 PMC3530905

[87] Cingolani, P. et al. A program for annotating and predicting the effects of single nucleotide polymorphisms, SnpEff. *Fly* **6**, 80–92 (2012). 22728672 10.4161/fly.19695 PMC3679285

[88] Baselga Cervera, B. Data from: stochastic phenotypic switching arises in response to directional selection in experimentally evolved multicellular yeast. Zenodo, 10.5281/zenodo.15652984 (2025). 10.1038/s42003-025-09414-9 PMC12855194 41455746

[89] Rueden, C. T. et al. ImageJ2: ImageJ for the next generation of scientific image data. *BMC Bioinform.* **18**, 529 (2017). 10.1186/s12859-017-1934-z PMC5708080 29187165

[90] Schindelin, J. et al. Fiji: an open-source platform for biological-image analysis. *Nat. Methods* **9**, 676–682 (2012). 22743772 10.1038/nmeth.2019 PMC3855844

[91] R: The R Project for Statistical Computing. https://www.r-project.org/.

[92] Pastore, M. & Calcagnì, A. Measuring distribution similarities between samples: a distribution-free overlapping index. *Front. Psychol.* **10**, 1089 (2019). 31231264 10.3389/fpsyg.2019.01089 PMC6558420

[93] Patro, R., Duggal, G., Love, M. I., Irizarry, R. A. & Kingsford, C. Salmon provides fast and bias-aware quantification of transcript expression. *Nat. Methods* **14**, 417–419 (2017). 28263959 10.1038/nmeth.4197 PMC5600148

[94] Chen, Y., Chen, L., Lun, A. T. L., Baldoni, P. L. & Smyth, G. K. edgeR v4: powerful differential analysis of sequencing data with expanded functionality and improved support for small counts and larger datasets. *Nucleic Acids Res.* **53**, gkaf018 (2025). 39844453 10.1093/nar/gkaf018 PMC11754124

[95] Ritchie, M. E. et al. limma powers differential expression analyses for RNA-sequencing and microarray studies. *Nucleic Acids Res.* **43**, e47 (2015). 25605792 10.1093/nar/gkv007 PMC4402510

[96] Hunter, J. D. Matplotlib: a 2D graphics environment. *Comput. Sci. Eng.* **9**, 90–95 (2007).

[97] Waskom, M. L. seaborn: statistical data visualization. *J. Open Source Softw.* **6**, 3021 (2021).

[98] Ge, S. X., Jung, D. & Yao, R. ShinyGO: a graphical gene-set enrichment tool for animals and plants. *Bioinformatics* **36**, 2628–2629 (2020). 31882993 10.1093/bioinformatics/btz931 PMC7178415

