# Supplementary material for: Stochastic phenotypic switching arises in response to directional selection in experimentally evolved multicellular yeast
Source: Commun Biol. 2025 Dec 27;9:134. doi: 10.1038/s42003-025-09414-9 (PMC12855194; doi:10.1038/s42003-025-09414-9)
Supplement: Supplementary file 1 — Supplemental Material [file 42003_2025_9414_MOESM1_ESM.pdf]

Suppl. Materials of: *Stochastic phenotypic switching arises in response to directional selection in experimentally evolved multicellular yeast.*

Beatriz Baselga-Cervera[12*], Nahui Olin Medina-Chávez[1], Noah Gettle[4] & Michael Travisano[123].

[1] Department of Ecology, Evolution and Behavior, University of Minnesota, St Paul, Minnesota 55108, United States.
[2] Minnesota Center for Philosophy of Science, University of Minnesota, Minneapolis, Minnesota 55455, United States.
[3] The Biotechnology Institute, University of Minnesota, St Paul, Minnesota, 55108, United States.
[4] Department of Biological Sciences, University of Denver, Denver, Colorado, 80208-9010, United States.

*Correspondence: bbaselga@umn.edu;beabaselga@gmail.com

Beatriz Baselga-Cervera ORCID: 0000-0003-3423-4780
Noah Gettle ORCID: 0000-0003-3407-4086
Nahui Olin Medina-Chávez ORCID: 0000-0003-3977-4233
Michael Travisano ORCID: 0000-0001-8168-0842

Data availability statement

All data described or analyzed in this study are included in the main text, SI Appendix, and raw data. Raw data can be found in the Zenodo repository DOI: 10.5281/zenodo.15652984.

RNASeq raw sequences will be released under the NCBI-GenBank. BioProject PRJNA1214970 accession number after publication. Genome reference *Saccharomyces cerevisiae* Y55 used in the analysis can be found under GCA_903819135.2 accession number and BioSample SAMEA6932012.

Acknowledgments

This work was supported by the Fundación Alfonso Martín Escudero [to B.B.C.] and John Templeton Foundation Grant ID 63455 granted to Michael Travisano and C. Ken Waters. We acknowledge the UMN Genomics Center for technical assistance with the RNA extractions.

Supplementary videos description

Supplementary Movie 1. Experimentally evolved multicellular yeast movie clip between 17 and 20 hours of growth (C1W8.1-derived strain) — video clip highlighting two instances of single-cell propagule generation from a multicellular cluster. The time-lapse video captures growth dynamics over this period, highlighting with the black box, the formation of single-cell propagule from a multicellular cluster on two occasions (visible in the upper left region of the frame). Images were acquired every 15 minutes using a 10x objective lens. See full time-lapse video (Suppl. Video 1) of in the Zenodo repository DOI: 10.5281/zenodo.15652984. Additional C1W8.1 strain videos (Suppl. Video 3 and 4).

Supplementary Movie 2. *ace2Δ knockout* constructed strain growth — video clip of a single large multicellular cluster. The video clip shows a single large multicellular cluster fragmenting into two large multicellular clusters at ~ 13 hours of growth and generating two small ancestral-like propagules at ~19 hours of growth. Microphotographs were obtained at 3-minute intervals under a 10x objective over 26 hours. See full 26-hours video (Suppl. Video 2) the Zenodo repository DOI: 10.5281/zenodo.15652984. Additional constructed strains videos (Suppl. Video 5 and 6).

Supplementary Movie 3. Experimentally evolved multicellular yeast growth over 24 hours (C1W8.1-derived strain) — cell division stops in small ancestral-like phenotypes. The footage captures multiple large multicellular clusters undergoing fragmentation into propagules. Additionally, a small ancestral-like cluster is observed undergoing division during the first 2 to 3 hours, followed by a cessation of division for the remainder of the time-lapse (visible in the lower left region of the frame within the square box). This early division phase is evident during the first 10 seconds of the video. Images were acquired every 5 minutes using a 10x objective lens.

Supplementary Figures

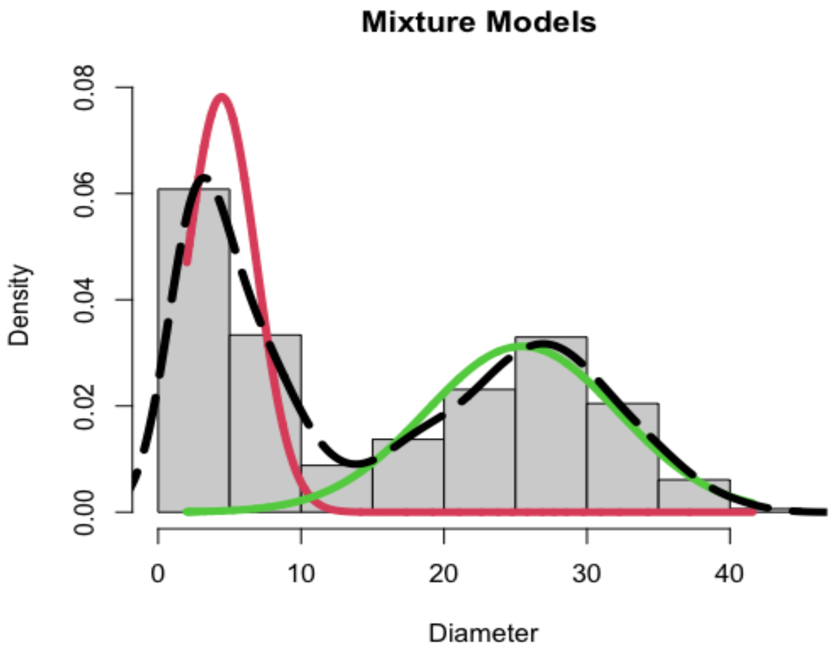

Fig. S1. Coulter Counter size distributions two-component normal mixture model plot. C1W8.2 derived strain diameter distribution analyses to obtain profiled subpopulations at 24 hours (red and green lines). Measured by an electronic particle counter (Multisizer 4 Coulter Counter® (Beckman Coulter).

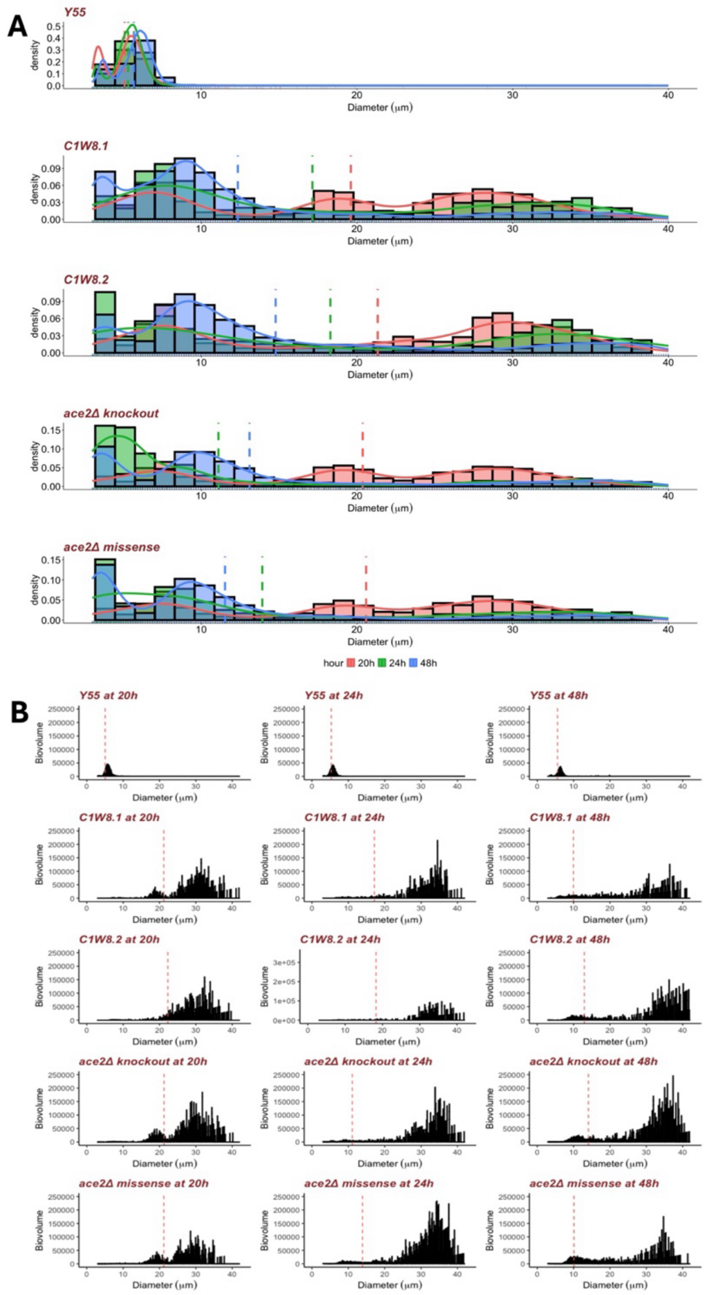

Fig. S2. Populations' particle size distributions. Measured by an electronic particle counter (Multisizer 4 Coulter Counter® (Beckman Coulter) represented as density (A) and biovolume (B) by population's diameter (μm). Each density— note different y-axis limits— and biovolume distribution depicts populations after 20h, 24h, and 48h growth in YPD media under shaken conditions of the five independent isolates merged. Vertical lines indicate mean diameter (μm). Strains: *Saccharomyces cerevisiae* ancestral unicellular Y55 strain, evolved strains C1W8.1 and C1W8.2 are multicellular derived genotypes, and *ace2Δ missense* (ACE2$^{c.1934A>T}$) and *ace2Δ knockout* constructed strains. Biovolume was calculated as the number of particles within each diameter range by the volume (μm3). Coulter Counter principle measures particle volume by detecting the displacement of fluid when a particle passes through a sensing zone. The instrument then calculates the equivalent spherical diameter of the particle based on its measured volume.

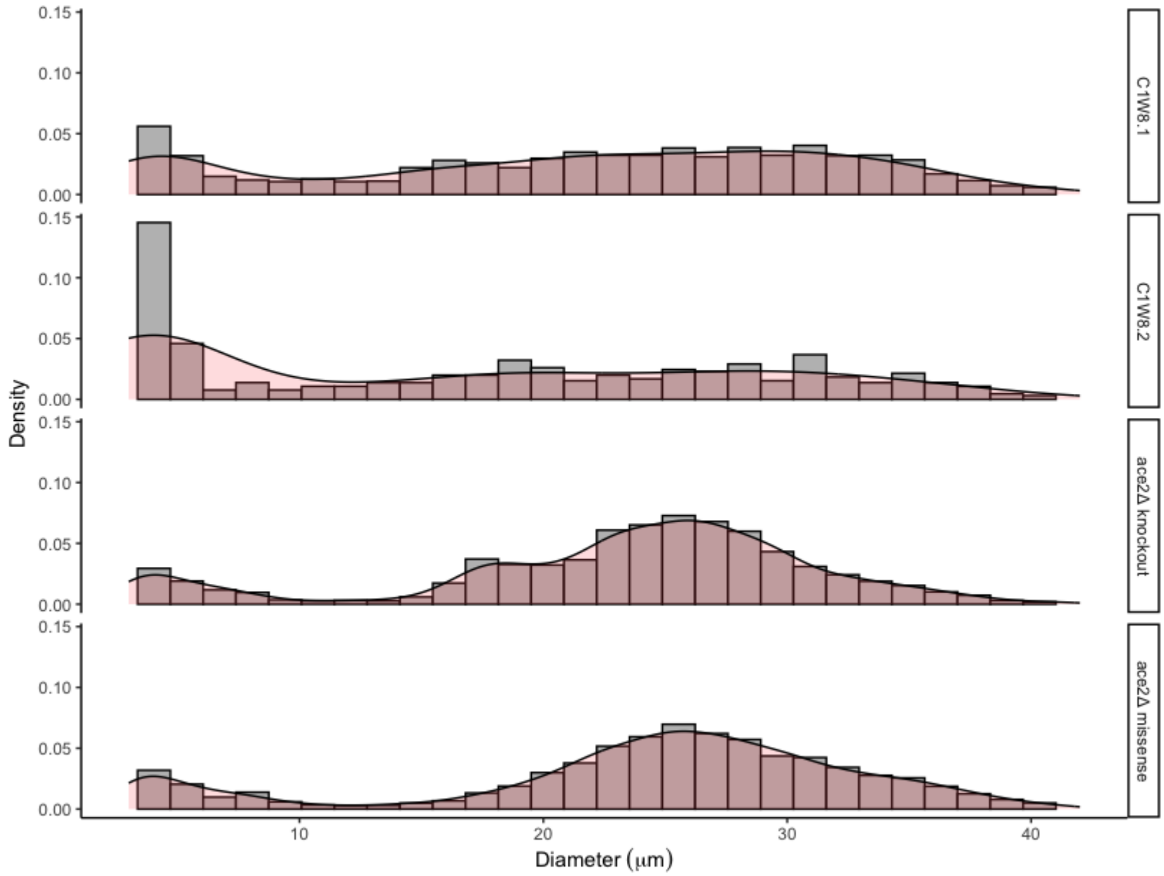

Fig. S3. Populations' particle size distributions at 12-hours growth in YPD. Particle size distributions measured by an electronic particle counter (Multisizer 4 Coulter Counter® (Beckman Coulter) are represented as density by population's diameter (µm). Strains: Saccharomyces cerevisiae evolved strains C1W8.1 and C1W8.2 are multicellular derived genotypes, and *ace2Δ missense* (ACE2 $^{c.1934\ A>T}$) and *ace2Δ knockout* constructed strains.

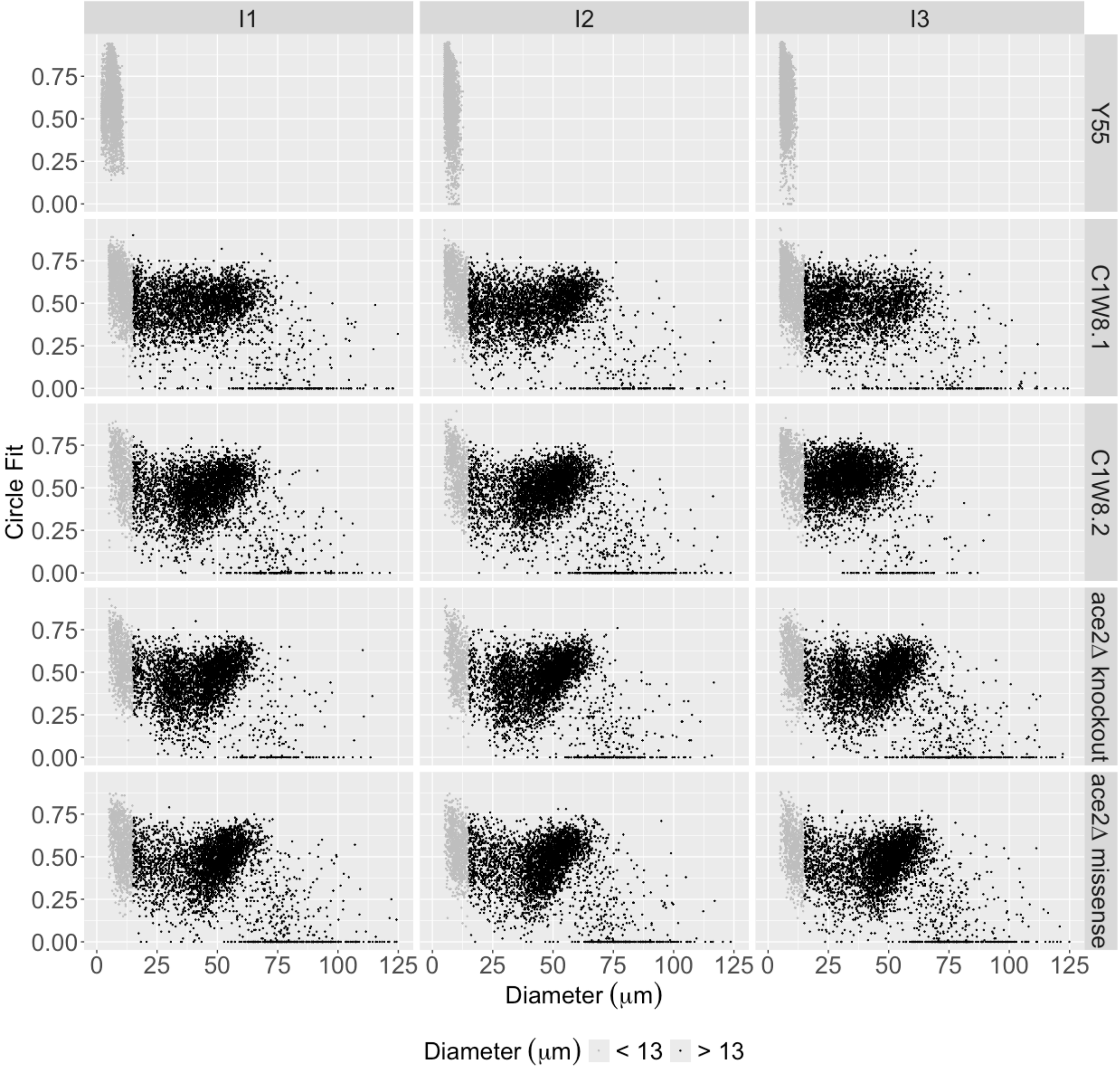

Fig. S4. Imaged particle size distributions. Scatterplot of the particle's images obtained with a digital flow cytometer (FlowCam® 3.0 Fluid Imaging Technologies). Circle Fit indicates the deviation of the particle from a best-fit circle. The color legend indicates those particles above and below 13 μm in diameter. The 13 μm diameter cutoff represents the maximum diameter value obtained in the Multisizer 4 Coulter Counter® runs of Y55 strain at 24-hours growth. Plot represents three isolates (I1, I2, and I3) per strain; *Saccharomyces cerevisiae* ancestral unicellular Y55 strain, evolved strains C1W8.1 and C1W8.2 are multicellular derived genotypes, and *ace2Δ missense* (ACE2 $^{c.1934\ A>T}$) and *ace2Δ knockout* constructed strains.

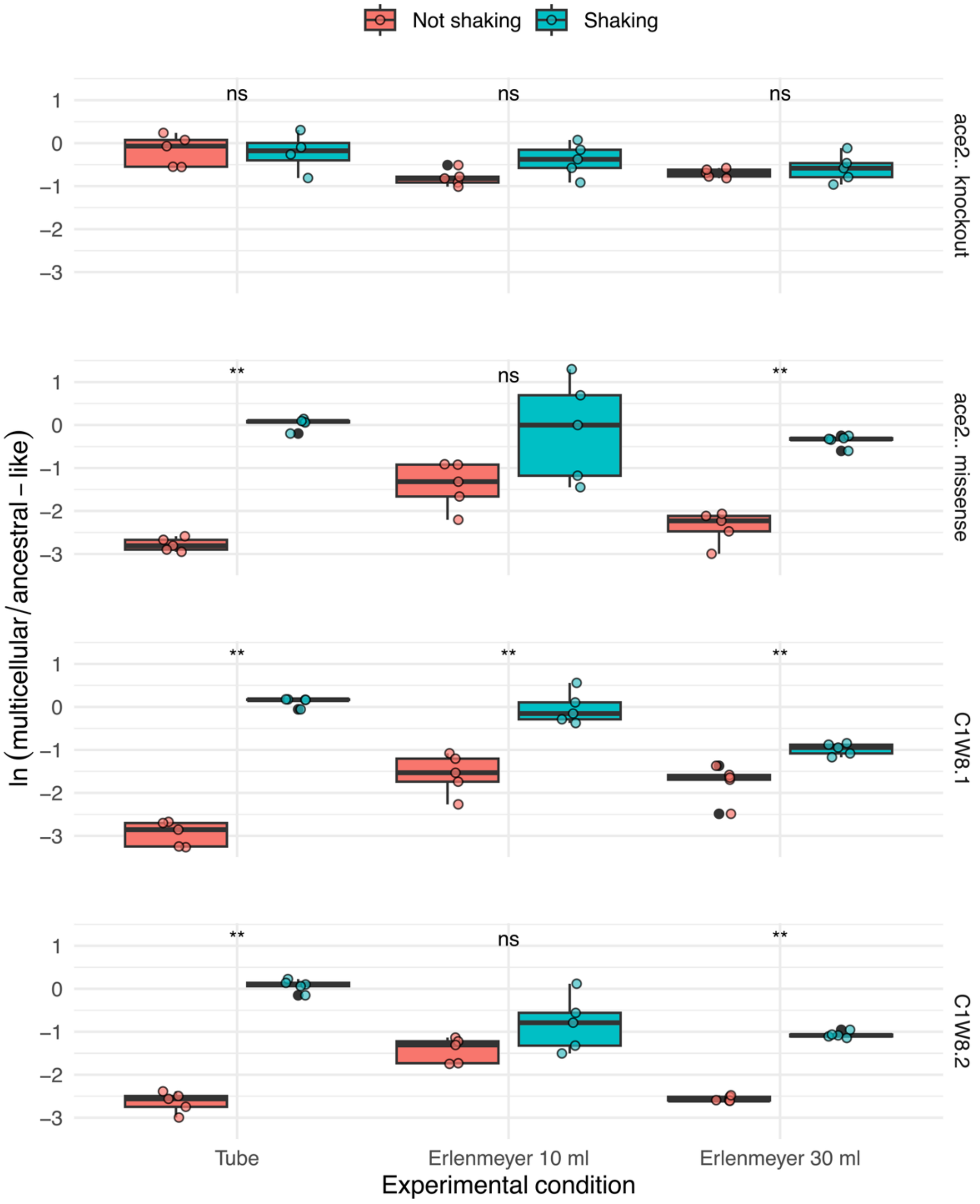

Fig. S5. Boxplots of the ln(multicellular clusters/ancestral-like) ratio under controlled environmental conditions. We observe an increase in unicellular-like phenotypes under non-shaking conditions across ecological conditions and strains. T-test paired measurements results are indicated with ** for $P < 0.01$ and ns for no significance. One isolate per strain was grown under six different experimental conditions: in culture tubes with 10 ml of media, 50 ml Erlenmeyer flasks with 10 ml and 30 ml of media, under non-shaking and shaking conditions at 250 rpm. Cultures were imaged under the microscope after 24 hours of growth at 30°C and analyzed by the ImageJ software. Particles were classified based on area as multicellular clusters and ancestral-like phenotypes. A size threshold of 7000 squared pixels was set as a cutoff for classification, the largest size found in the unicellular ancestor. Refer to the Zenodo repository for raw and analyzed images, and ImageJ-generated measurements (DOI: 10.5281/zenodo.15652984).

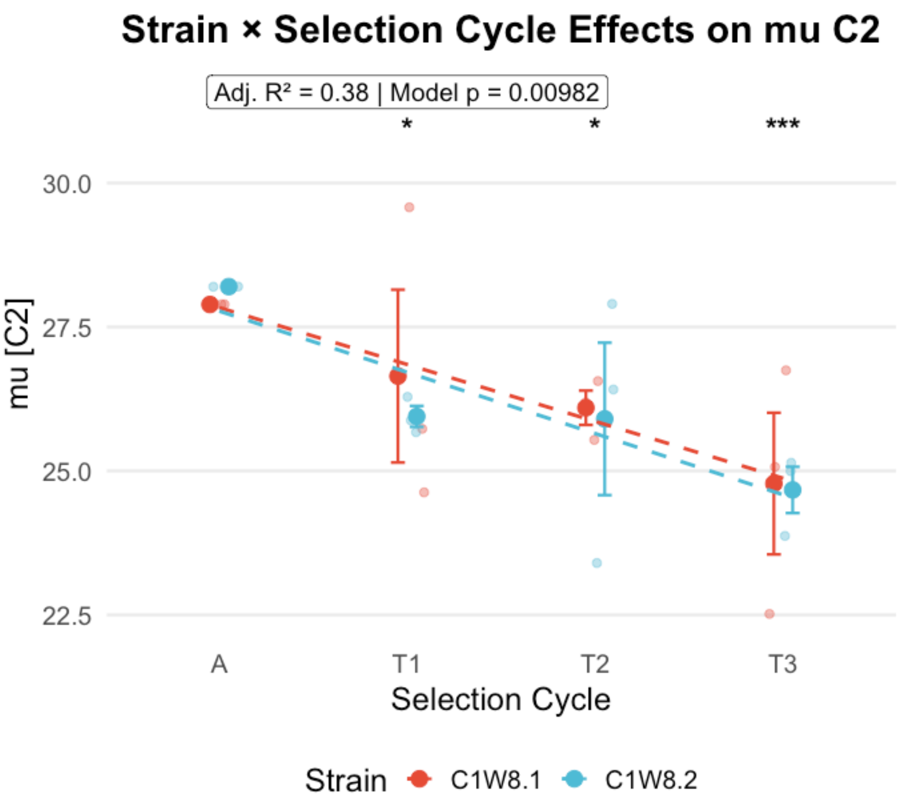

Fig. S6. Mean ± SE (mu) for the C2 component corresponding to large multicellular clusters over selection cycles. We observe a decrease in mean multicellular cluster diameter (µm) over selection cycles in both derived strains (C1W8.1 and C1W8.2). mu[C2] was obtained using the R package mixtools/normalmix. Data represent three independent lineages per strain. We observe a significant effect of selection cycle in mu[C2] (linear regression model: mu[C2] ~ Strain + selection cycle, $F_{4,19} = 4.52$, $p < 0.01$). A= ancestral.

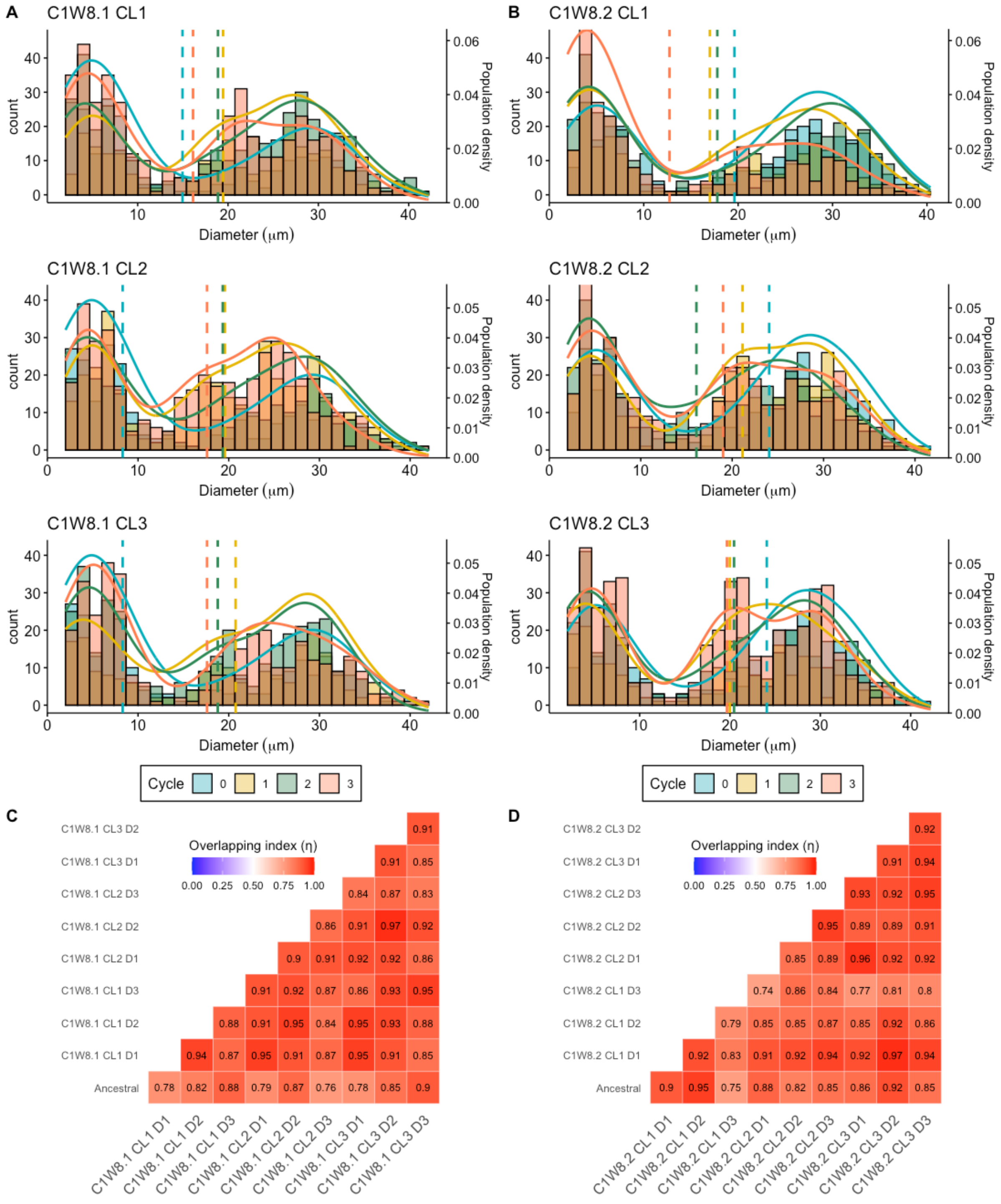

Fig. S7. Population size distributions over the selection experiment. Selection consisted of plating the top fraction of the population after gravitational selection. (A) and (B) Diameter distributions (µm) of the particle size counts and densities of six colony lineages (CL) (three of each for evolved strain, C1W8.1 and C1W8.2) over selection cycles. Colors represent the cycle of selection. (C) C1W8.1 and (D) C1W8.2 heatmaps of the overlapping index (η) pairwise comparisons of the ancestral and selected lineages (D= selection cycle). Measured by an electronic particle counter (Multisizer 4 Coulter Counter® (Beckman Coulter).

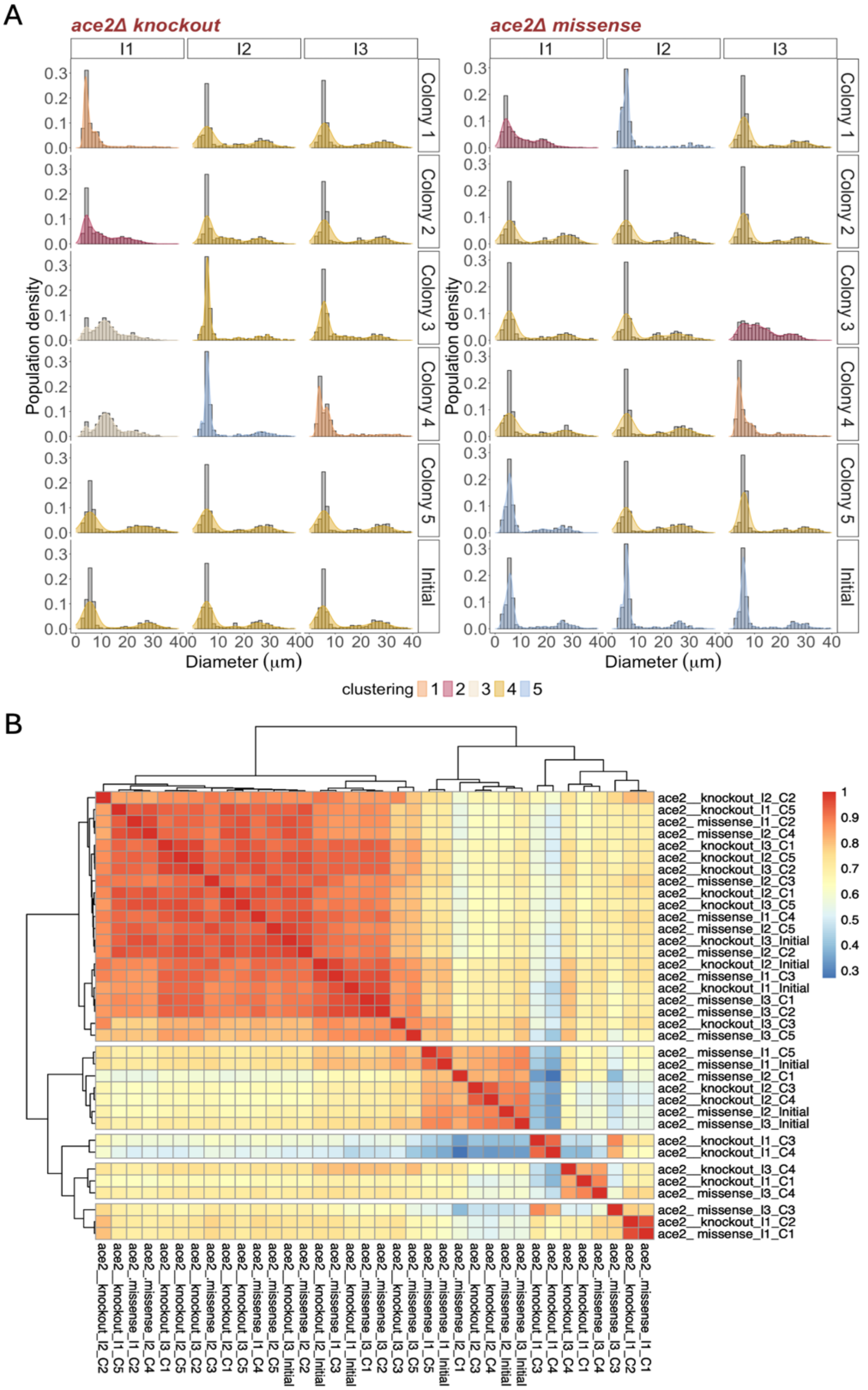

Fig. S8. Population size distributions of individual colonies and initial populations of ace2Δ missense and *ace2Δ knockout* strains. (A) Population density plots of whole populations before (initial) gravitational selection and of five individual colonies randomly picked (C1 to C5) from the Top fraction of the population per *ace2Δ knockout* and *ace2Δ missense* isolate (isolates I1, I2 & I3). (B) Heatmap comparisons of the overlapping index (η) of the diameter frequency distributions. Dendrograms denote hierarchical agglomerative clustering using Ward's minimum variance method (Ward.D2). Isolates clustered in five groups.

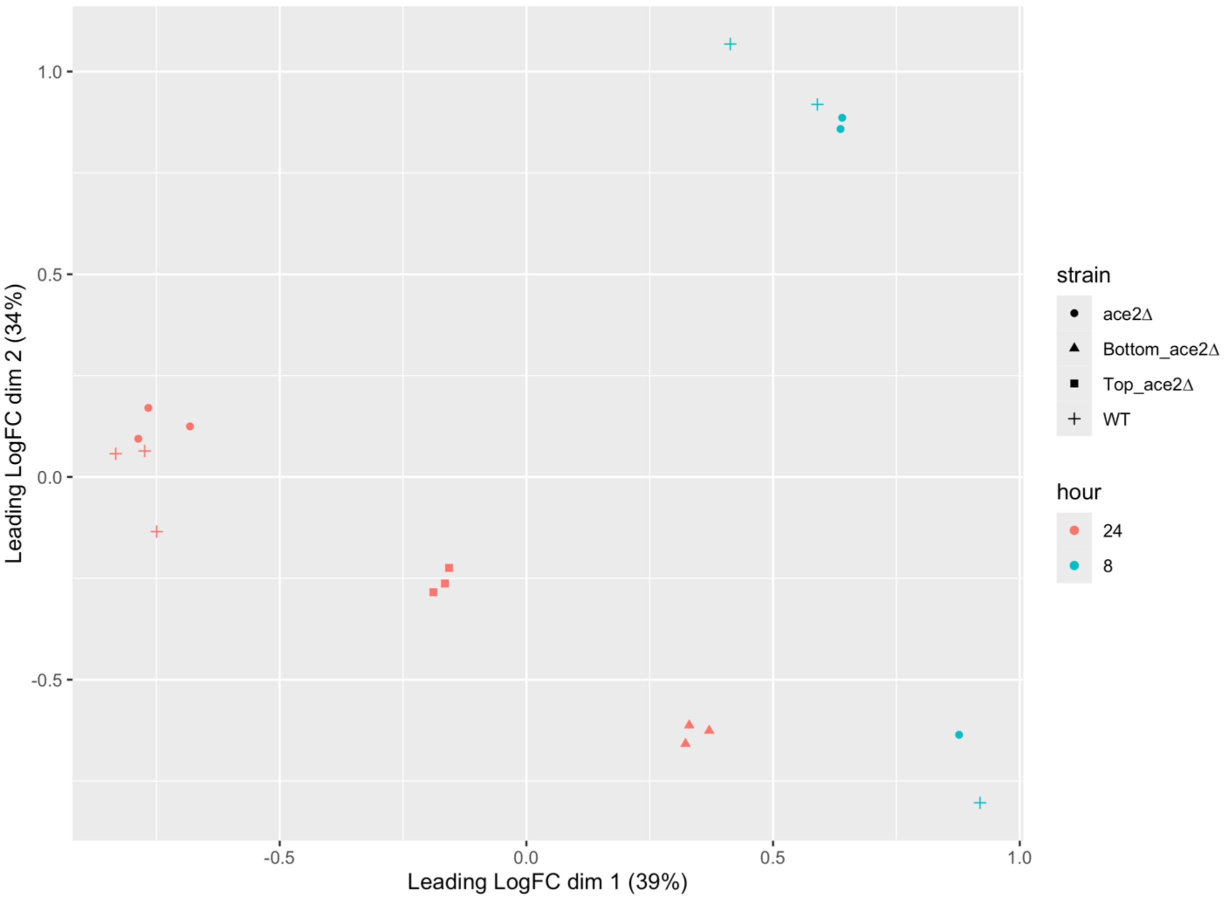

Fig. S9. Multidimensional Scaling plot of the RNA expression profiles. Plot shows the average absolute size of the leading log2-fold-changes (LogFC) of *Saccharomyces cerevisiae* ancestral unicellular wildtype (WT) Y55 strain and *ace2Δ missense* constructs at 8 (blue) and 24-hours (red) growth, compared to the *ace2Δ missense* constructs Top and Bottom subdistributions expression profiles at 24-hours growth.

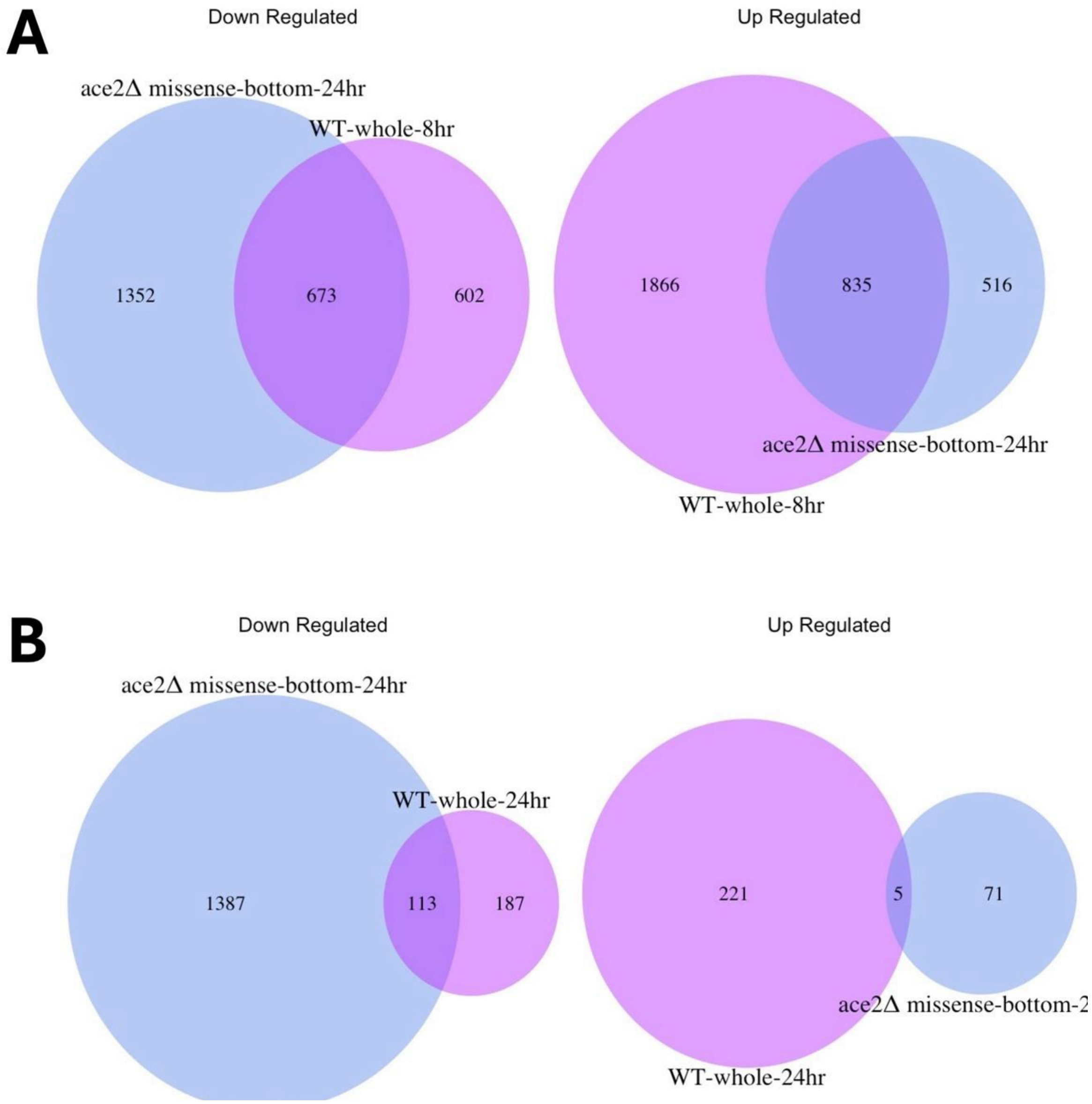

Fig. S10. Venn diagrams of the rank–rank hypergeometric analysis to compare differentially expressed populations. Diagrams showing the number of upregulated and downregulated genes in genes from *ace2Δ missense* Bottom vs WT populations at 8 (A) and 24-hours (B) growth.

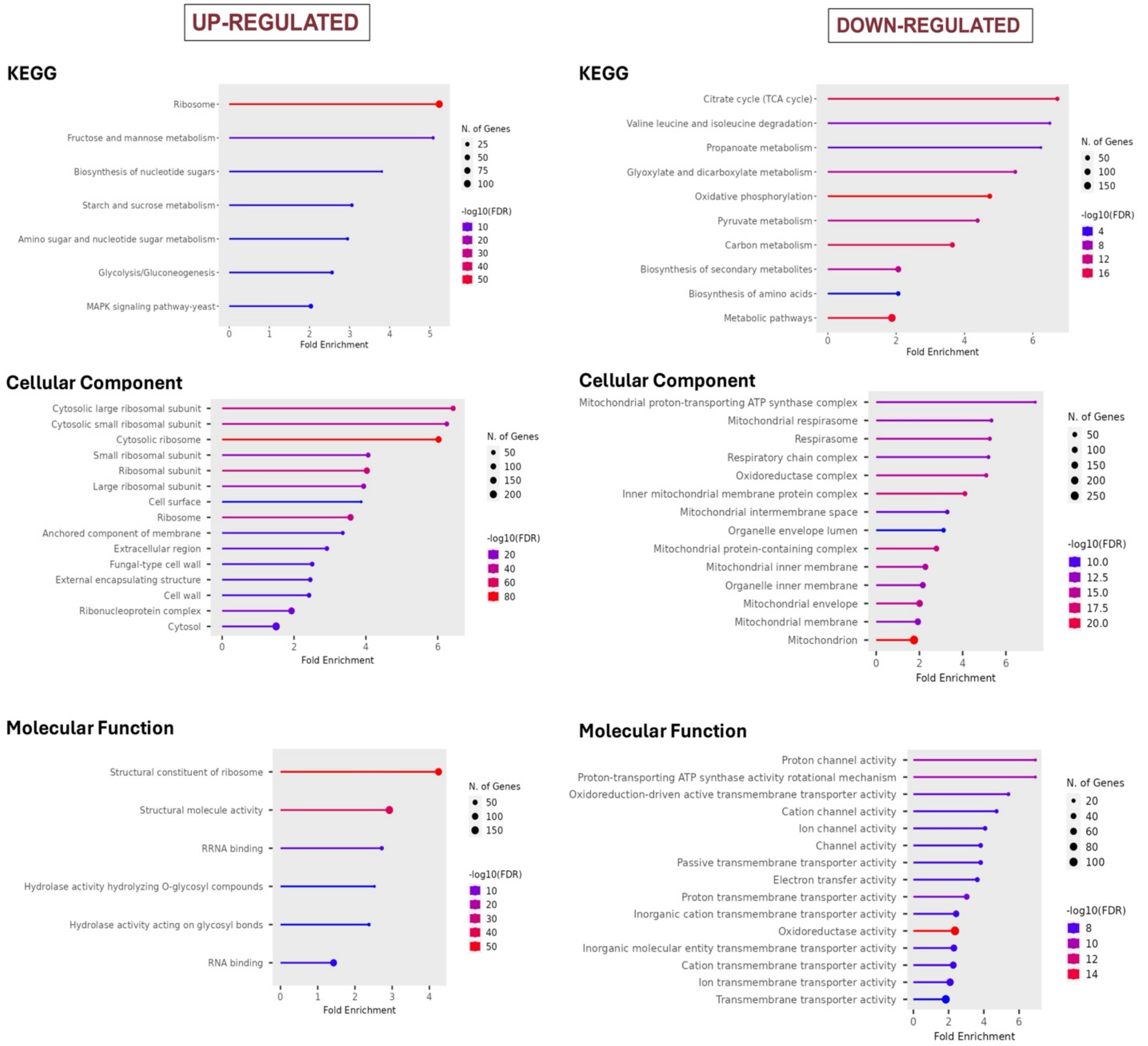

Fig. S11. Gene ontology (GO) enrichment analyses of the expression profiles of the constructed *ace2Δ missense* isogenic strain subdistributions after gravitational selection. The top subdistribution was considered the control, and the bottom subdistribution the experimental condition. Up-regulated (left) and Down-regulated (right) KEGG (Kyoto Encyclopedia of Genes and Genomes), GO cellular components and GO Molecular function terms obtained using ShinyGO.

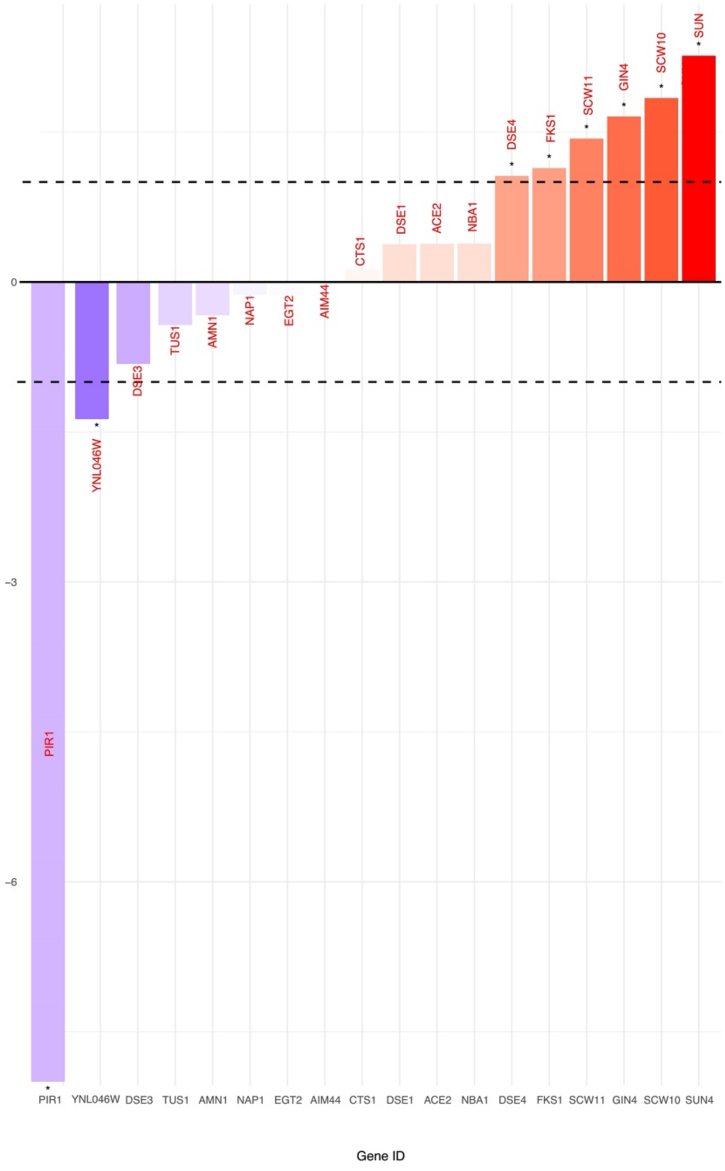

Fig. S12. Expression of genes previous identified as causal loci in the evolution of multicellular clusters between Top and Bottom subpopulations. No systematic pattern of gene expression differences wer observed for ACE2, AIM44, AMN1, CTS1, DES1, EGT2, NAP1, and NBA1 between the two subpopulations. While TUS1, DSE3, and YNL046W were downregulated in the multicellular size class, DES4, GIN4, FKS1, SCW10, SCW11, and SUN4 were upregulated in the bottom subpopulation composed of multicellular clusters. Ace2p is responsible for controlling post-division separation of mother and daughter cells following mitosis, and the rest of the genes are tied to Ace2p transcriptional regulation. Y-axis represents -log2 fold-change (Log2FC).

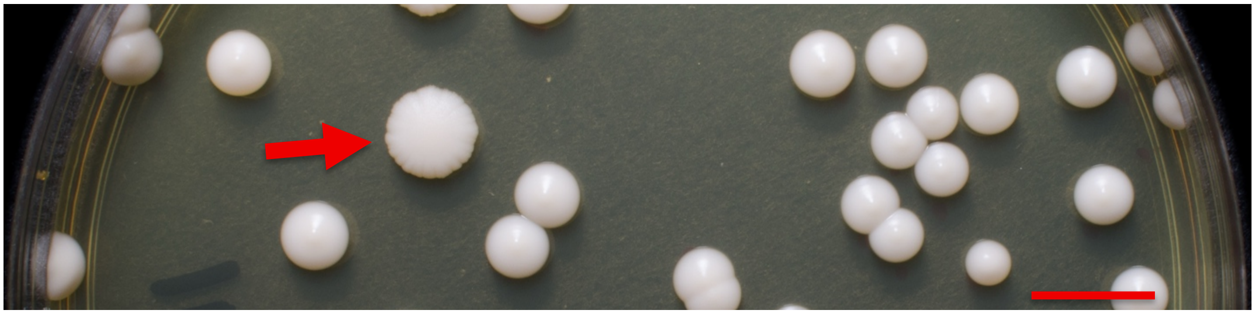

Fig. S13. Colony phenotypes of the snowflake yeast system. Multicellular *Saccharomyces cerevisiae* strains form macroscopically larger colonies characterized by a rugose edge and a wrinkled surface texture (indicated by red arrow), in contrast to the smooth, circular colonies formed by wild-type strains also present on the plate. The image shows a 100 × 15 mm Petri dish containing YPD agar, incubated at 30 °C for 72 hours. Scale bar 10 mm.

Supplementary Tables

Table S1. Descriptive statistics of the population's diameter (µm) distributions at 24h growth. Values were obtained using an electronic particle sizer (Coulter Counter Multisizer4, Beckman®). Hartigan's dip test (D value) indicated that all empirical distributions were at least bimodal. Two modes were identified in all multicellular populations, except *ace2Δ knockout* isolate 1, which presented four modes. Refer to the Zenodo repository for the descriptive statistics of all the strains at 12-, 20-, 24-, and 48-hour growth descriptive stats in 10 ml YPD culture tubes at 250 rpm and 30 °C (DOI: 10.5281/zenodo.15652984).

Strain	Isolate	N	Mean	SD	Median	MAD	D value	p-value
**Y55**	1	5323	5.31	0.95	5.44	0.69	0.041	< 0.001
	2	4585	5.26	0.76	5.28	0.69	0.045	< 0.001
	3	6563	5.22	0.96	5.28	0.69	0.042	< 0.001
	4	7437	5.23	0.92	5.28	0.69	0.044	< 0.001
	5	5918	5.22	0.91	5.28	0.69	0.042	< 0.001
	**Average**		5.25	0.90	5.31	0.69		
**C1W8.1**	1	731	17.14	11.16	12.16	9.27	0.049	< 0.001
	2	747	18.19	11.69	12.78	9.03	0.055	< 0.001
	3	1102	14.11	11.49	8.88	7.41	0.043	< 0.001
	4	1656	14.34	10.54	9.03	7.18	0.034	< 0.001
	5	639	19.54	12.03	19.97	17.84	0.098	< 0.001
	**Average**		16.66	11.38	12.56	10.15		
**C1W8.2**	1	356	18.29	13.03	12.63	13.67	0.085	< 0.001
	2	437	22.85	12.07	28.09	12.51	0.067	< 0.001
	3	603	23.69	11.76	29.19	9.96	0.061	< 0.001
	4	379	21.62	11.98	20.91	17.37	0.054	< 0.001
	5	540	22.55	12.75	28.41	13.20	0.087	< 0.001
	**Average**		21.80	12.32	23.84	13.34		

ace2Δ knockout	1	1698	11.10	10.38	5.91	3.71	0.036	< 0.001
	2	1228	15.10	11.89	9.50	8.34	0.036	< 0.001
	3	703	15.25	12.19	9.19	8.11	0.050	< 0.001
	4	1025	14.25	12.23	8.25	7.18	0.049	< 0.001
	5	1147	13.22	11.64	7.78	6.49	0.053	< 0.001
	**Average**		13.79	11.66	8.13	6.76		
ace2Δ missense	1	2023	13.92	11.44	8.72	7.41	0.035	< 0.001
	2	917	14.36	11.72	8.88	8.11	0.043	< 0.001
	3	1159	14.61	12.03	8.72	7.88	0.055	< 0.001
	4	1028	15.58	12.25	9.66	9.27	0.055	< 0.001
	5	1639	13.75	11.84	7.94	6.72	0.044	< 0.001
	**Average**		14.45	11.86	8.78	7.88		

Table S2. Non-Gaussian bimodal distributions of evolved and constructed strains, two-component normal mixture models at 24 hours of growth. Individual peak information was obtained using the R package mixtools/normalmix. Profiled subpopulations are Component 1 (C1) (lower diameter range, unicellular subgroup) and Component 2 (C2) (upper diameter range, multicellular subgroup). *Lambda* refers to the final mixing proportions assigned to each component (% of the population). *mu* and *Sigma* are each component's means and standard deviations

Strain	Isolate	Lambda C1	Lambda C2	mu C1	mu C2	Sigma C1	Sigma C2	Loglik
**C1W8.1**								
	1	0.54	0.46	7.90	27.95	2.62	6.73	-2522.76
	2	0.66	0.34	5.80	28.87	2.67	6.64	-5457.54
	3	0.70	0.30	7.08	30.62	3.23	5.38	-3678.62
	4	0.66	0.34	7.39	27.76	2.95	5.97	-5538.40
	5	0.49	0.51	7.79	30.61	3.11	4.36	-2178.09
**C1W8.2**								
	1	0.59	0.41	7.94	33.00	4.47	3.60	-1245.14
	2	0.41	0.59	9.33	32.09	4.38	4.61	-1562.09
	3	0.40	0.60	10.58	32.58	5.93	3.52	-2129.32
	4	0.53	0.47	11.40	33.02	5.81	4.28	-1392.68
	5	0.45	0.55	9.19	33.30	4.90	3.83	-1911.39
**ace2Δ knockout**								
	1	0.74	0.26	5.72	26.63	2.25	8.86	-5232.76
	2	0.64	0.36	7.11	29.58	3.16	7.31	-4257.90
	3	0.65	0.35	6.88	30.64	3.04	6.20	-2397.09
	4	0.66	0.34	6.06	30.04	2.80	6.60	-3443.77
	5	0.35	0.65	3.59	18.40	0.48	11.46	-3791.89
**ace2Δ missense**								
	1	0.68	0.32	6.72	29.30	3.08	6.55	-6835.83
	2	0.65	0.35	6.49	29.20	3.10	6.38	-3130.16
	3	0.66	0.34	6.49	30.29	3.18	5.54	-3923.81
	4	0.62	0.38	6.68	30.22	3.31	5.81	-3563.63
	5	0.66	0.34	5.80	28.87	2.67	6.64	-5457.54

Table S3. Statistical analyses of the ratio ln (multicellular clusters/unicellular-like). One isolate per strain was grown in culture tubes with 10 ml of media, 50 ml Erlenmeyer flasks with 10 mL and 30 mL of media, under non-shaking and shaking conditions at 250 rpm. YPD media was used across all the conditions. Cultures were assessed after 24-hours of growth at 30°C. Strains: Saccharomyces cerevisiae ancestral unicellular Y55 strain, evolved strains C1W8.1 and C1W8.2 are multicellular derived genotypes, and ace2 missense ($ACE2^{c.1934\ A>T}$) and ace2 knockout constructed strains. ANOVA (ln(multicellular clusters/unicellular like) ~ strain * vessel * shaking_condition).

	Df	Sum Sq	Mean Sq	F value	Pr(>F)	
**strain**	3	14	4.67	29.694	1.24E-13	***
**vessel**	2	4.58	2.29	14.574	3.02E-06	***
**shaking_condition**	1	58.87	58.87	374.684	< 2e-16	***
**strain: vessel**	6	4.47	0.75	4.746	0.000286	***
**strain: shaking_condition**	3	15.42	5.14	32.713	1.30E-14	***
**vessel:shaking_condition**	2	8.58	4.29	27.304	4.28E-10	***
**strain:vessel:shaking_condition**	6	7.08	1.18	7.508	1.34E-06	***
**Residuals**	95	14.93	0.16			

*** P < 0.001

Table S4. Statistical analyses of derived strain distributions over three cycles of selection. Three independent lineages per derived strains (C1W8.1 and C1W8.2) were selected by plating the top fraction of mixed populations after gravitational selection (CL = cycle of selection). Population diameter (μm) distributions were obtained using an electronic particle sizer (Coulter Counter Multisizer4, Beckman®). Means in a column followed by the same letter are not significantly different (Wilcoxon method, P > 0.05).

**Strain (lineage)**	**Mean diameter (μm)**		
**C1W8.1**	**CL1**	**CL2**	**CL3**
**ancestral**	15.52a	…	
**1**	19.86a	18.72b	19.37b
**2**	19.31b	17.99bc	17.70bc
**3**	16.64b	17.05ac	16.91ac
**K-W chiSquare**	19.32	12.92	10.42
**p-value**	<0.001	<0.001	<0.05
**C1W8.2**			
**ancestral**	20.09a	…	
**1**	17.58b	19.76a	18.41b
**2**	18.34b	16.48b	18.23b
**3**	13.45c	17.55b	18.15b
**K-W chi**	52.56	28.78	11.04
**p-value**	<0.001	<0.001	<0.05

Table S5. Two-component normal mixture model analyses of derived strain distributions over three selection cycles. Individual peak information was obtained using the R package mixtools/normalmix. Profiled subpopulations are Component 1 (C1) (lower diameter range, unicellular subgroup) and Component 2 (C2) (upper diameter range, multicellular subgroup). *Lambda* refers to the final mixing proportions assigned to each component (% of the population). *mu* and *Sigma* are each component's means and standard deviations.

**Strain**	**Cycle of selection**	**Lambda C1**	**Lambda C2**	**mu C1**	**mu C2**	**Sigma C1**	**Sigma C2**	**Loglik**
**C1W8.1**								
	**Ancestral**	0.57	0.43	6.01	27.89	2.44	5.64	-785.38
	Lineage 1							
	1	0.30	0.70	5.94	25.73	1.95	6.13	-798.98
	2	0.34	0.66	5.38	26.56	2.12	6.43	-1431.08
	3	0.43	0.57	5.65	25.07	2.18	6.27	-1465.72
	Lineage 2							
	1	0.31	0.69	5.82	24.63	1.88	6.19	-1257.93
	2	0.40	0.60	5.57	26.19	2.21	6.38	-931.17
	3	0.32	0.68	5.34	22.52	1.68	6.40	-1432.68
	Lineage 3							
	1	0.76	0.24	16.18	29.58	10.57	1.66	-762.43
	2	0.40	0.60	5.74	25.54	2.25	5.99	-1225.33
	3	0.47	0.53	6.02	26.75	2.10	5.81	-1164.12
**C1W8.2**								
	**Ancestral**	0.63	0.37	6.32	28.20	2.44	5.19	-1075.71
	Lineage 1							
	1	0.42	0.58	5.42	26.29	2.00	5.53	-745.86
	2	0.42	0.58	5.37	27.90	2.04	5.70	-1140.92
	3	0.55	0.45	5.04	23.87	1.68	7.18	-845.55
	**Lineage 2**							
	1	0.30	0.70	5.52	25.88	1.90	5.81	-1169.38
	2	0.38	0.62	5.23	23.40	1.74	6.95	-1181.16
	3	0.39	0.61	5.92	25.00	2.30	6.35	-1336.48
	**Lineage 3**							
	1	0.35	0.65	5.08	25.67	1.88	5.82	-717.05
	2	0.39	0.61	5.37	26.41	2.04	5.88	-1087.56
	3	0.36	0.64	5.68	25.14	2.00	5.91	-1622.13

Table S6. Primers used in this study

Name	Target Locus	Template	Sequence
ACE2null_F	ACE2	pFAa6-kanMX6	CAAAGAAATCTATAGGACCAAAAACGGTG TTAATACAATCCGTACGCTGCAGGTCGAC
ACE2null_R	ACE2	pFAa6-kanMX6	ATTATTTACTATGTTAATATCATGCATAGA TAAATGTTCGATCGATGAATTCGAGCTCG
ACE2wtmx_fusn_F	ACE2	pFAa6-kanMX6	ATTTCTTTACGATTTACGTACACTGTAGTCT TAAGGGCCACGTACGCTGCAGGT
ACE2wtmx_fusn_R	ACE2	pFAa6-kanMX6	ACCAAAGGATGTGTGAAGCTGGTTTGTAGT AGTTAAAGGGATCGATGAATTCGA
ACE2_R	ACE2	chrXII	TGGGGTTTTAGATCGTGTCTC
ACE2__F	ACE2	chrXII	CGTTGCAGGGAGACTCAA
ACE2cnf_F	ACE2	chrXII	ATGGGAGCGTCCTAATGAAA

Appendix A: Mathematical model

1.- Model summary:

$$\frac{dM}{dt} = \alpha log_2(M) + \eta(t) + \gamma U \quad (1)$$

$$\frac{dU}{dt} = S(t)\,\beta M - \gamma U \quad (2)$$

$$S(t) \sim Bernoulli\ (\frac{1}{1+e^{-k\,(M-\theta)}}) \quad (3)$$

where,

M: large multicellular clusters.
U: smaller unicellular-like clusters.
α: *M* growth rate coefficient of large clusters individuals (not cellular division).
β: rate at which *U* derives from *M*.
γ: rate at which *U* derives into *M*.
t: time
η(t): random fluctuations in *M* growth.
S(t): represents the phenotypic switching event as a discrete random variable (0 to 1) that modulates U growth, modeled as a Bernoulli random variable.
θ: represents the threshold for the phenotypic switch event adjusted as a function of *k*.
k: controls for the steepness of the sigmoid. Higher *k* = more abrupt transition.

2.- Model description:

This is a differential equations model that incorporates stochasticity and a probabilistic phenotypic switch. The model represents two phenotypic populations (*M* and *U*) over time in which *M* grows at its own dynamics, and *U* cannot exist independently of *M*. *M* growth is dynamic, controlled by *η(t)*. *η(t)* is a stochastic term that introduces random fluctuations in *M* growth (i.e., developmental noise) by generating a random value from a normal distribution with a mean of 0 and a standard deviation of 0.1. *U* is generated by *M* at rate *β*. *S(t)* represents a stochastic binary switch (0 or 1), indicating whether the switch from *M to U* occurs at time *t*. The switching probability follows a sigmoidal (logistic) function of *M*.

3.-Key assumptions:

-*M* growth, define as cluster fragmentation into two clusters not cellular division, is logarithmic (in base 2), presenting density-dependent effects. This type of growth is sublinear and only positive when *M*>1. This reflects a system where growth slows down dramatically at high densities, and there is no growth when U ≤ 1.
-*U* is only generated by *M*. *U* does not reproduce, define as generating new individuals of either phenotype (small ancestral-like or large multicellular clusters). *U* cannot exist independently of *M*. *U* accumulates over time.

-*U* transition to *M* is controlled by γ, representing the possible feedback loop.
-We assume that neither *M* nor *U* die or decay, representing a yeast population in exponential growth and post-diauxic phase.
-The switching probability increases with *M*. Lag period requires *M* to grow large enough before generating *U*. Before θ, *U* stays constant (or zero).

4.- Modeling parameters:

In our model, the initial state was set as c(M = 200, U = 200), dt = 1 run on a sequence (0, 2880, by = 1), the threshold (θ) was set at M = 13,000 and η was set at 0.1 (equivalent to 10% noise).

5. Model Tables and Figures

Table M1. Experimental data for the differential equations model. Percentage values were calculated using two-component normal mixture models from size distribution's values of *ace2Δ missense* (ACE2 $^{c.1934\ A>T}$) and *ace2Δ knockout* constructed strains subpopulations at 12, 20, 24 and 48 hours-growth. Phenotypes: unicellular-like (*U*) and multicellular clusters (M).

**Strain (isolate)**	**12 hours**		**20 hours**		**24 hours**		**48 hours**	
ace2Δ knockout	*% U*	*% M*	*% U*	*% M*	*% U*	*% M*	*% U*	*% M*
1	10.87	89.13	32.65	67.35	73.84	26.16	77.16	22.84
2	11.46	88.54	23.69	76.31	64.14	35.86	80.82	19.18
3	13.11	86.89	30.99	69.01	64.71	35.29	78.86	21.14
ace2Δ missense								
1	14.70	85.30	25.11	74.89	68.04	31.96	87.54	12.46
2	12.77	87.23	31.43	68.57	65.21	34.79	82.34	17.66
3	14.81	85.19	24.18	75.82	65.59	34.41	86.28	13.72

Table M2. Root Mean Squared Error (RMSE) analyses of the model and experimental values where γ = 0. Phenotypic dynamics differences between the mathematical model's predicted values and experimental values. Experimental values of *ace2Δ missense* (ACE2 $^{c.1934\ A>T}$) and *ace2Δ knockout* constructed strains subpopulations at 12, 20, 24 and 48 hours-growth. γ represents the feedback loop probability that *U* transition into *M* over *t*.

Model Reference	steepness (*k*)	alpha	beta	RMSE_Total
k0.001_a1_b0.005	0.001	1	0.005	8.60
k0.1_a1_b0.005	0.1	1	0.005	8.98
k0.01_a1_b0.005	0.01	1	0.005	9.03
k5_a1_b0.005	5	1	0.005	9.03
k5_a1_b0.01	5	1	0.01	17.59
k0.1_a1_b0.01	0.1	1	0.01	17.59
k0.01_a1_b0.01	0.01	1	0.01	17.66
k0.001_a1_b0.01	0.001	1	0.01	18.29
k0.001_a0.5_b5	0.001	0.5	5	19.70
k0.001_a0.5_b0.1	0.001	0.5	0.1	28.49

Table M3. Root Mean Squared Error (RMSE) analyses of the model and experimental values where γ range between 0.001 to 0.1. RMSE values are higher when $\gamma > 0$.

**Model Reference**	**steepness (*k*)**	**alpha**	**beta**	**gamma**	**RMSE_Total**
k0.001_a1.188_b0.005_g0.001	0.001	1.188	0.005	0.001	16.615
k1.667_a1.188_b0.005_g0.001	1.667	1.188	0.005	0.001	16.759
k05.000_a1.188_b0.005_g0.001	5.000	1.188	0.005	0.001	16.759
k2.223_a1.188_b0.005_g0.001	2.223	1.188	0.005	0.001	16.759
k3.889_a1.188_b0.005_g0.001	3.889	1.188	0.005	0.001	16.759
k4.445_a1.188_b0.005_g0.001	4.445	1.188	0.005	0.001	16.759
k3.334_a1.188_b0.005_g0.001	3.334	1.188	0.005	0.001	16.759
k1.112_a1.188_b0.005_g0.001	1.112	1.188	0.005	0.001	16.760
k0.556_a1.188_b0.005_g0.001	0.556	1.188	0.005	0.001	16.760
k2.778_a1.188_b0.005_g0.001	2.778	1.188	0.005	0.001	16.760

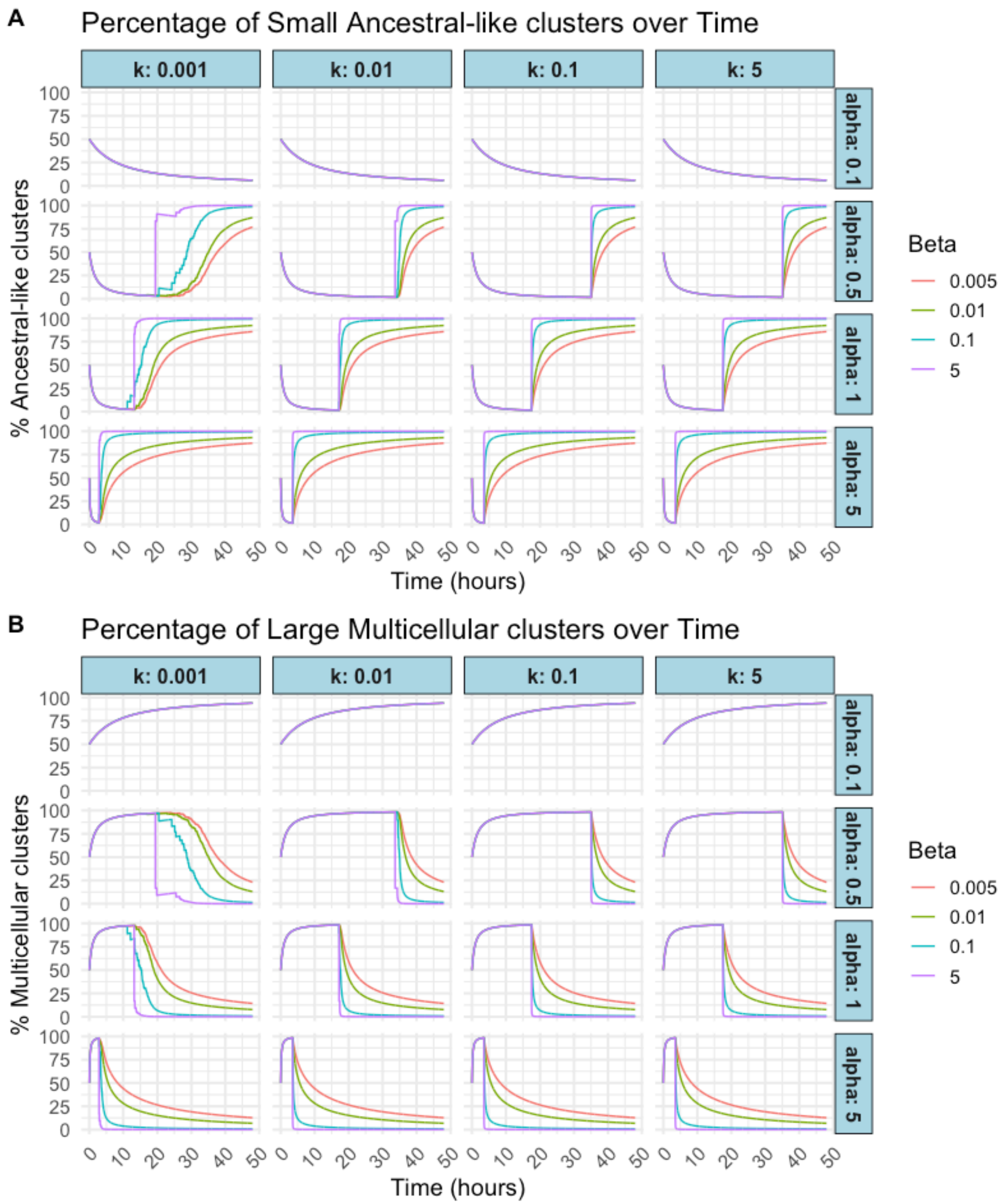

Fig. M1. Phenotypic dynamics obtained from the differential equations model under different simulations. Time series represent the percentage of small ancestral-like clusters (A) and large multicellular clusters (B) by steepness of the sigmoid function (*k*), small clusters generation rate (beta) and multicellular clusters growth rate (alpha). The probability of small clusters transitioning into large clusters (gamma) is 0. Note: only four parameters for each variable, k, alpha and beta are shown.

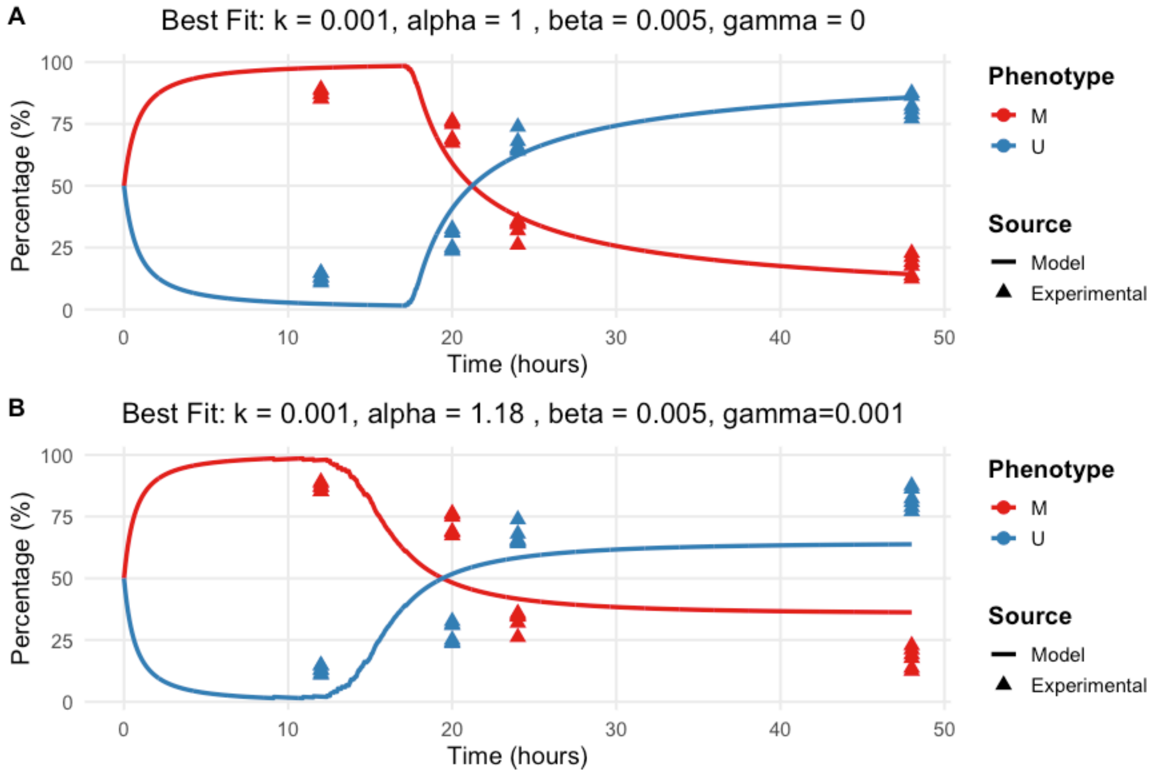

Fig. M2. Best-fit models plotted with the experimental data where (A) $\gamma = 0$ and (B) $\gamma > 0$. Time series represent the percentage of small ancestral-like (blue) and large multicellular clusters (red) phenotypes of the model (lines) and experimental data (triangles) over 48-hours.
