# Supplementary material for: Stochastic phenotypic switching arises in response to directional selection in experimentally evolved multicellular yeast
Source: Commun Biol. 2025 Dec 27;9:134. doi: 10.1038/s42003-025-09414-9 (PMC12855194; doi:10.1038/s42003-025-09414-9)
Supplement: Supplementary file 2 — Description of Additional Supplementary Materials [file 42003_2025_9414_MOESM2_ESM.pdf]

Description of Additional Supplementary Files

File name: Supplementary Movie 1

Description: C1W8.1 from 17 to 21 hours growth

File name: Supplementary Movie 2

Description: ace2KO from 12.5 to 19 hours growth

File name: Supplementary Movie 3

Description: C1W8.1 from 24 hours growth
